# Sequential Conversion of *D*-Xylose to Furfuryl Alcohol by Bet:FA:MA–Water Dehydration and EutG–GDH Whole-Cell Bioreduction

**DOI:** 10.3390/biology15181652

**Published:** 2026-09-18

**Authors:** Haoyu Chai, Jutao Li, Cuiluan Ma, Yu-Cai He

**Affiliations:** 1 School of Life Sciences, Hubei University, Wuhan 430062, China; 202421107011782@stu.hubu.edu.cn (H.C.); l19956408336@163.com (J.L.); 2 School of Pharmacy & School of Biological and Food Engineering, Changzhou University, Changzhou 213164, China

**Keywords:** betaine:formic acid:malonic acid, aldehyde reductase, furfural, furfuryl alcohol, chemobiological catalysis

## Abstract

A sequential two-stage chemo-biocatalytic process was investigated for the conversion of *D*-xylose to furfuryl alcohol (FOL), an important furan derivative widely used in the manufacture of resins, polymers, fuels, and other value-added products. In the first stage, *D*-xylose was dehydrated to furfural (FAL) in a ternary Betaine:Formic acid:Malonic acid–water (Bet:FA:MA–H_2_O) reaction medium. Under the selected conditions, a FAL yield of 65.8% was obtained from 22.5 g/L *D*-xylose using 15 wt% Bet:FA:MA at 170 °C for 30 min. For the subsequent bioreduction stage, a recombinant *Escherichia coli* strain co-expressing the aldehyde reductase EutG and glucose dehydrogenase (GDH) was constructed. Using glucose as a co-substrate, EutG–GDH whole cells were able to reduce commercial FAL at concentrations up to 150 mM under the selected conditions of 40 °C and pH 7.5. For the sequential process using the *D*-xylose-derived dehydration liquor, the acidic reaction mixture was adjusted to pH 7.5 and diluted from 98.7 to 30.0 mM FAL before whole-cell bioreduction. The conditioned 30.0 mM FAL feed was essentially completely converted to FOL within 24 h. These results demonstrate the laboratory-scale feasibility of sequentially combining Bet:FA:MA–H_2_O-mediated *D*-xylose dehydration with EutG–GDH whole-cell FAL reduction after interstage pH adjustment and dilution.

## Simple Summary

Furfuryl alcohol is an important bio-based chemical widely used in the manufacture of resins, polymers, fuels, and other value-added products. In this study, a sequential chemical and biological strategy was investigated for the conversion of *D*-xylose, a sugar derived from lignocellulosic biomass, into furfuryl alcohol. In the first stage, a ternary betaine:formic acid:malonic acid–water (Bet:FA:MA–H_2_O) reaction medium was used for the dehydration of *D*-xylose to furfural. In the subsequent biological stage, recombinant *Escherichia coli* cells co-expressing the aldehyde reductase EutG and glucose dehydrogenase (GDH) were used to reduce furfural to furfuryl alcohol. The acidic dehydration liquor required pH adjustment and dilution before whole-cell bioreduction. Overall, this study demonstrates the laboratory-scale feasibility of sequentially combining Bet:FA:MA–H_2_O-mediated *D*-xylose dehydration with EutG–GDH whole-cell furfural reduction and provides a basis for further optimization of the two-stage process.

## 1. Introduction

With the deterioration of the environment and the reduction in fossil-fuel reserves, increasing attention has been directed toward the development of renewable and sustainable energy [1,2,3,4,5]. Lignocellulosic biomass has attracted considerable attention because of its renewable, abundant, carbon-containing feedstock and non-food lignocellulosic resource. Thermochemical pretreatment can solubilize or depolymerize the hemicellulose fraction of biomass [6,7]. In the acid hydrolysis process, the hemicellulose components in biomass can be transformed into *D*-xylose and then further processed into compounds such as furfural (FAL) [8]. FAL is an important biomass-derived platform chemical used for the production of solvents, resins, fuels, and numerous value-added furan derivatives [9,10,11].

Acid catalysis is usually used to produce FAL from *D*-xylose. However, traditional acid catalysis may produce harmful gases and a large amount of waste liquid containing acid, which will cause serious pollution to the environment [12,13,14,15]. Different from traditional ionic liquids, deep eutectic solvents (DESs) have the advantages of environmental protection, economy, easy preparation, recovery, and high solubility [16,17]. DESs are eutectic solvents of binary and ternary systems mainly composed of hydrogen-bond donors (HBDs) and hydrogen-bond acceptors (HBAs) [18,19]. In recent years, DES (CA:Betaine)-H_2_O catalyzed the conversion of carbohydrates (60 g/L) in corn cob to FAL (74.6 mM), formic acid (96.5 mM), and glucose (40.3 mM) for 30 min at 170 °C [20]. DESs have attracted interest because of their simple preparation, tunable physicochemical properties, low volatility, and potential recyclability. However, their environmental performance depends strongly on their individual components and process conditions.

Furfuryl alcohol (FOL) is the major hydrogenation product of FAL and one of the most important biomass-derived furan chemicals [21,22,23]. The global production of furfural currently exceeds 300 kt per year, and more than two-thirds of the produced furfural is further converted into FOL, indicating a global FOL production and consumption scale of more than 200 kt per year. At the industrial scale, FOL is produced predominantly through the catalytic hydrogenation of FAL with molecular hydrogen in either the liquid or vapor phase. Copper-based catalysts, particularly copper chromite, have historically been employed for this transformation, while Cr-free Cu-, Ni-, Co-, and other heterogeneous catalysts are increasingly being investigated to improve environmental compatibility, selectivity, and catalyst stability [23]. Therefore, conventional catalytic hydrogenation of FAL remains the dominant technology responsible for the largest share of commercial FOL production.

Asia, particularly China, represents the major production region because of its large furfural manufacturing capacity and well-developed furan-chemical industry [21]. In addition, TransFurans Chemicals operates one of the largest dedicated FOL production facilities in Europe, with an annual production exceeding 40,000 metric tons [23]. The largest current application of FOL is the manufacture of furan and polyfurfuryl alcohol resins, particularly as no-bake binders for foundry molds [24]. It is also used in corrosion-resistant materials, refractory products, adhesives, coatings, wood modification, and as a chemical intermediate for the synthesis of tetrahydrofurfuryl alcohol and other furan derivatives [23,24,25]. Looking forward, increasing demand for renewable carbon materials is expected to expand the use of FOL in bio-based thermosetting resins, sustainable composites, modified wood, bio-solvents, and fuel- or chemical-oriented furan derivatives [24,25]. Consequently, the development of more environmentally benign and energy-efficient routes for FOL production from lignocellulosic biomass remains of considerable industrial interest.

**Figure 1:**
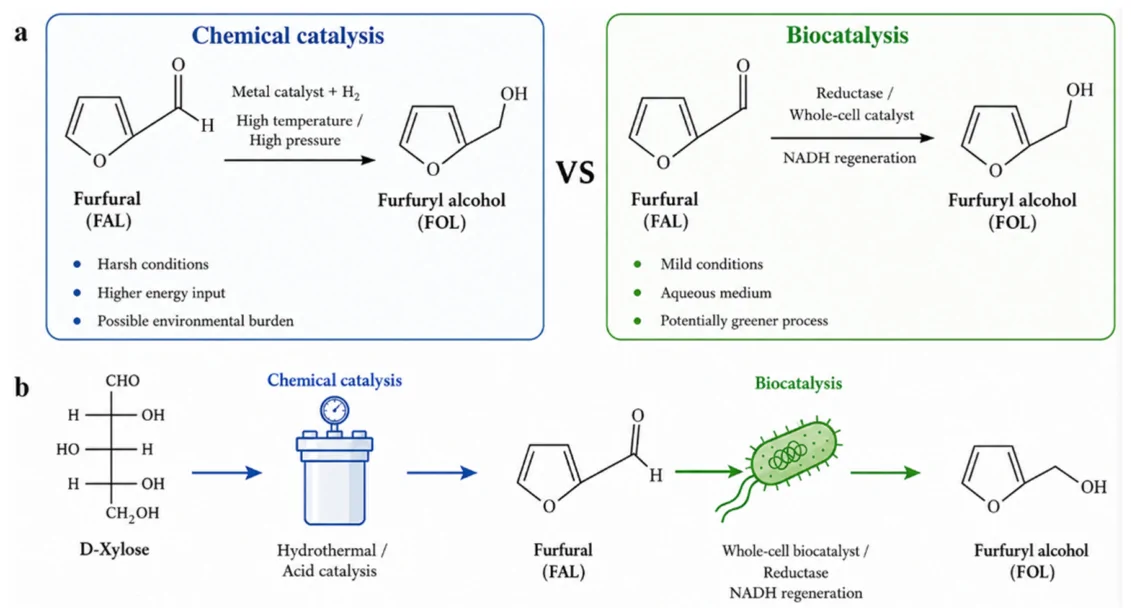
Types of catalysis for the synthesis of FOL from FAL (**a**); The sequential two-stage chemo-biocatalytic conversion of *D*-xylose to FOL investigated in this study (**b**). In panel (**a**), blue and green elements distinguish chemical catalysis and biocatalysis, respectively, while black arrows indicate the direction of reaction; In panel (**b**), blue arrows indicate the chemical-catalysis stage, whereas green arrows indicate the biocatalytic stage.

Conventional chemical hydrogenation of FAL generally requires pressurized H_2_ and heterogeneous metal catalysts and may involve elevated temperature and pressure [23]. In contrast, biocatalytic reduction offers an alternative under comparatively mild aqueous conditions and can avoid high-pressure H_2_ and metal hydrogenation catalysts, although catalyst loading, cofactor regeneration, substrate toxicity, and downstream processing remain important limitations (Figure 1a). Through bioreduction with CG-19 cells (pH 7.5, 35 °C, 24 h), 150 mM FAL can be transformed into FOL in 75% yield [22]. *Bacillus* cells can also transform 52 mM FAL into FOL at pH 7.0 and 50 °C, achieving a FOL yield of 73.0% within 24 h [26].

Recently, considerable interest has focused on the production of FOL from renewable and inexpensive lignocellulosic biomass [27,28]. DESs can function as reaction media and, in some systems, as catalysts [29,30,31,32,33]. The DES composed of toluenesulfonic acid monohydrate, ferric chloride, and choline chloride can convert FAL derived from hemicellulose to butyl levulinate (BL) with a yield of 98% [32]. The acidic DES of choline chloride and sulfamic acid can dehydrate fructose to 5-hydroxymethylfurfural (5-HMF), and the yield of 5-HMF can reach 90% [30]. The recombinant *E. coli* DCF can convert FAL derived from *D*-xylose to FOL in Betaine:Benzenesulfonic acid-H_2_O (BE:BA-H_2_O) [31]. In the Lactic acid:Betaine-H_2_O (LA:Bet-H_2_O) system, HMFOMUT cells can effectively convert FAL derived from *D*-xylose to FOL [33]. Despite advances in DES-mediated FAL production and whole-cell bioreduction, sequential integration of the two catalytic stages remains challenging because the acidic chemical reaction medium is not directly compatible with microbial biocatalysts. Residual organic acids, DES components, salts formed during neutralization, and FAL-derived compounds may also affect cell viability and intracellular cofactor regeneration. Therefore, improving interstage compatibility while maintaining efficient cofactor regeneration remains important for developing sequential chemo-biocatalytic routes for FOL production.

In the present study, a ternary betaine/formic acid/malonic acid (Bet:FA:MA) system was evaluated as a reaction medium for the dehydration of *D*-xylose to FAL. A recombinant *Escherichia coli* strain co-expressing the aldehyde reductase EutG and glucose dehydrogenase (GDH) was subsequently constructed, with glucose supplied as a co-substrate to support intracellular cofactor recycling during FAL reduction. The effects of ternary formulation composition and Bet:FA:MA loading, *D*-xylose concentration, reaction temperature and time, as well as the major parameters governing whole-cell reduction, were investigated. Finally, the chemical dehydration and biological reduction stages were combined as a sequential two-stage chemo-biocatalytic route for the production of FOL from *D*-xylose.

## 2. Materials and Methods

### 2.1. Materials

Furfural (FAL), furfuryl alcohol (FOL), *D*-xylose, betaine (Bet), formic acid (FA), malonic acid (MA), and other chemicals were all supplied by Aladdin Chemical Reagent Co., Ltd. (Shanghai, China).

### 2.2. Preparation of Ternary Reaction Media (Bet:FA:MA)

Six ternary formulations were prepared by mixing betaine (Bet) or choline chloride (ChCl) with glycerol (Gly), formic acid (FA), malonic acid (MA), and/or lactic acid (LA) in a 300 mL round-bottomed flask at 500 rpm with magnetic stirring at 80 °C. The mixtures were stirred until clear, homogeneous liquids were obtained. The resulting formulations were Bet:FA:MA, Bet:Gly:LA, Bet:MA:LA, ChCl:FA:MA, ChCl:Gly:LA, and ChCl:Gly:MA (1:1:1 mol:mol:mol). These ternary formulations were evaluated as reaction-medium additives/co-solvents in aqueous media.

### 2.3. Transformation of D-Xylose into FAL in Bet:FA:MA–Water Reaction Media

The effects of Bet:FA:MA loading (0–25 wt%), *D*-xylose concentration (100–800 mM), reaction temperature (160–180 °C), and reaction time (5–60 min) were investigated. For the selected condition used in the sequential process, 1.125 g of *D*-xylose, 7.5 g of Bet:FA:MA, and 41.375 g of deionized water were combined to give a total reaction mass of 50.0 g and reacted at 170 °C for 30 min. The mixture was then transferred to a 100 mL high-pressure reactor (TGYF-B, Shanghai Yaote Equipment Instrument Co., Ltd., Shanghai, China) and stirred at 500 rpm to dehydrate *D*-xylose into FAL. *D*-Xylose concentrations of 100, 150, 200, 300, 400, and 800 mM were investigated. All experiments were performed independently in triplicate, and results are expressed as mean ± standard deviation. The formed FAL was analyzed by HPLC. The FAL yield was calculated as below:

(1) $$\mathrm{FAL}\;\mathrm{yield}\;(\%)\;=\;\frac{FAL\;produced\;(mM)}{Initial\;D\mathrm{-xylose}\;(mM)}\times 100$$

### 2.4. Construction of Recombinant E. coli for Bioreduction of FAL to FOL

The aldehyde reductase (ALRs) EutG was from *E. coli* BL21(DE3). The gene encoding EutG was cloned into the pRSFDuet expression vector. A GDH module using glucose as the co-substrate was subsequently introduced to support intracellular cofactor recycling during FAL reduction. The GDH was derived from *Bacillus megaterium*. To achieve the co-expression of GDH and EutG in *E. coli* BL21(DE3), the construction process was as follows: The nucleotide sequences were provided in Table S1 (see Supplementary Files). The primers EutG-F and EutG-R were adopted to amplify the reductase EutG. The primers GDH-F and GDH-R were used to amplify GDH. The primers Duet-F and Duet-R were adopted to linearize the Duet vector, and the primer sequences were provided in Table S2 (see Supplementary Files). The amplified fragments were purified, and the purified ones were ligated with T5 and transferred to the competent *E. coli* Top10 cells. Positive clones were verified by DNA sequencing and then introduced into the competent cells of *E. coli* BL21. The *E. coli* EutG-GDH cells were inoculated onto an LB agar plate containing antibiotics (50 µg/mL kanamycin). The cells were cultivated and collected as previously reported [10].

The effects of reaction temperature, pH, metal ions, glucose loading, and substrate concentration on recombinant *E. coli* EutG-GDH whole-cell bioreduction were investigated using commercial FAL as substrate. The impacts of temperature (20–50 °C), pH (6.0–9.0), metal ions (K^+^, Cr^3+^, Ni^2+^, Ca^2+^, Sn^4+^, Zn^2+^, Mg^2+^, Fe^2+^, Mn^2+^ and Al^3+^) (1 mM), co-substrate and precursor concentrations on cell biocatalytic activity were tested. EutG-GDH (a wet-cell loading of 0.1 g/mL) and FAL (100 mM) were added into 5 mL tubes in a 2 mL system. After incubation on a shaking table (200 rpm) for a certain time, FAL (substrate) and FOL (product) were determined by HPLC. Unless otherwise stated, the standard reaction mixture contained 100 mM FAL, glucose at a glucose:FAL molar ratio of 2:1, recombinant *E. coli* EutG-GDH cells at a final wet-cell loading of 0.1 g/mL, and 100 mM phosphate buffer (pH 7.5) in a total volume of 2.0 mL. All experiments were performed independently in triplicate, and results are expressed as mean ± standard deviation. The FOL yield was calculated as below:

(2) $$\mathrm{FOL}\;\mathrm{yield}\;(\%)\;=\;\frac{FOL\;produced\;(mM)}{Initial\;FAL\;(mM)}\times 100$$

### 2.5. HPLC Analysis

HPLC (LC-2030C 3D, Shimadzu Corporation, Kyoto, Japan) was used with a Discovery^®^C18 (3.9 mm × 150 mm, 4 μm; Supelco, Bellefonte, PA, USA) column. FAL and FOL were eluted with 80 vol% water, 20 vol% methanol, and 0.10 wt% trifluoroacetic acid (TFA) as the mobile phase. The designed flow rate was 0.80 mL/min at 35 °C. FAL and FOL were detected at 254 and 210 nm, respectively (Figures S1 and S2, see Supplementary Files). Prior to HPLC analysis, reaction samples with initial FAL concentrations of 30–150 mM were serially diluted to an overall dilution factor of 150-fold, so that the concentrations of FAL and FOL fell within the validated calibration range of 0.2–1.0 mM. The original analyte concentrations were calculated by multiplying the concentrations determined from the calibration curves by the corresponding dilution factor.

## 3. Results and Discussion

### 3.1. Dehydration of D-Xylose to FAL in Ternary Reaction Media

**Figure 2:**
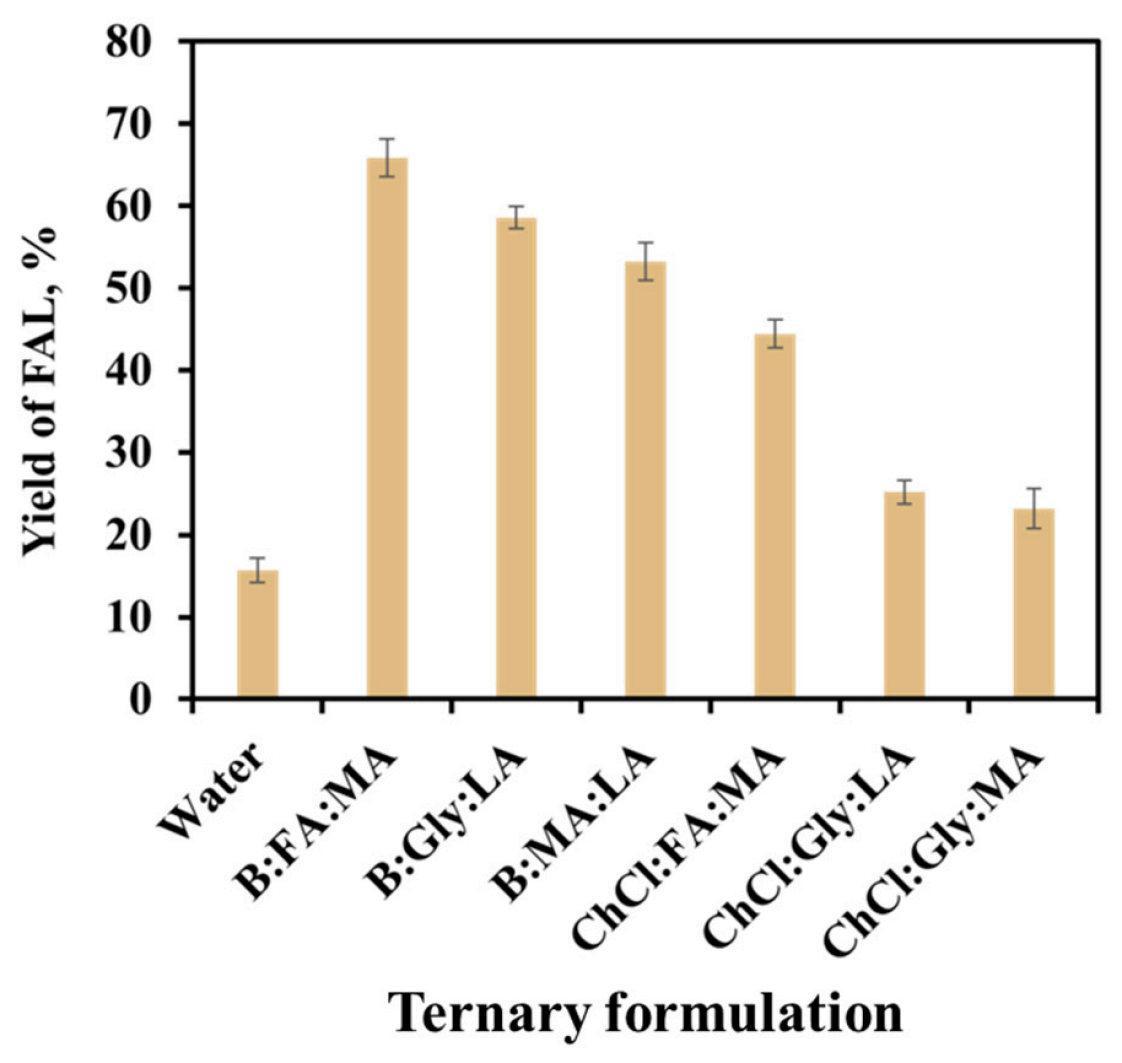
Effect of different ternary formulations (15.0 wt%) on FAL generation [150 mM *D*-xylose, 170 °C, 30 min]. Data are presented as mean ± standard deviation (SD) from independent experiments (*n* = 3).

FAL can be produced from hemicellulosic substrates through acid-catalyzed reactions, and conventional liquid-acid processes may involve challenges associated with catalyst separation and product recovery [34,35]. In this study, six ternary formulations, namely Bet:FA:MA, Bet:Gly:LA, Bet:MA:LA, ChCl:FA:MA, ChCl:Gly:LA, and ChCl:Gly:MA (1:1:1 mol:mol:mol), were evaluated as reaction media for the dehydration of *D*-xylose to FAL. Significant differences in FAL formation were observed among the tested formulations. At 170 °C for 30 min, Bet:FA:MA gave the highest FAL yield of 65.8% (Figure 2). Under the same conditions, Bet:Gly:LA and Bet:MA:LA afforded FAL yields of 58.6% and 53.2%, respectively, whereas ChCl:FA:MA gave a yield of 44.4%. The FAL yields obtained with ChCl:Gly:LA and ChCl:Gly:MA were approximately 25%. In the absence of the ternary formulation, the FAL yield was 16.7%.

**Figure 3:**
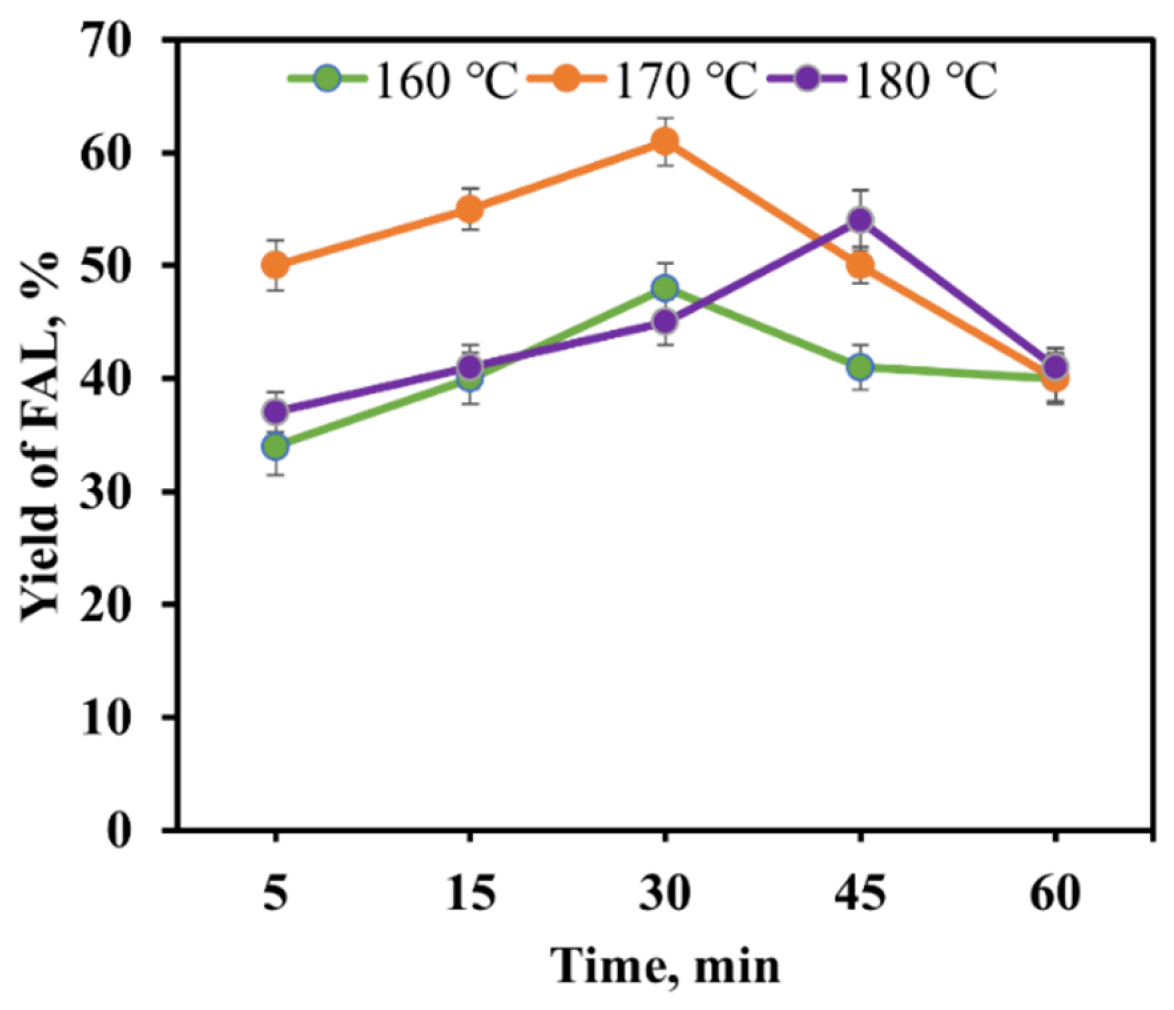
Effects of time (5–60 min) and temperature (160–180 °C) in Bet:FA:MA-H_2_O on FAL generation [200 mM *D*-xylose, Bet:FA:MA 15 wt%]. Data are presented as mean ± standard deviation (SD) from independent experiments (*n* = 3).

The effects of dehydration temperature and reaction time were subsequently investigated using the Bet:FA:MA–water reaction medium. As shown in Figure 3, the FAL yield increased when the reaction temperature was raised from 160 to 170 °C and then decreased at 180 °C. The lower FAL yield at higher temperature may be associated with secondary degradation and condensation reactions of FAL, including humin formation and other acid-catalyzed side reactions [36,37]. Accordingly, 170 °C gave the highest FAL yield among the tested temperatures and was selected for subsequent experiments. At 170 °C, increasing the reaction time from 5 to 30 min increased the FAL yield from 49.8% to 60.7%, whereas prolonging the reaction to 60 min decreased the yield to 40.3%. During prolonged *D*-xylose dehydration, FAL may undergo further degradation and condensation to form insoluble humins and soluble degradation products [38,39]. Because only a limited number of experimental points were examined, these observations should be regarded as empirical trends within the tested reaction range rather than as definitive kinetic relationships.

**Figure 4:**
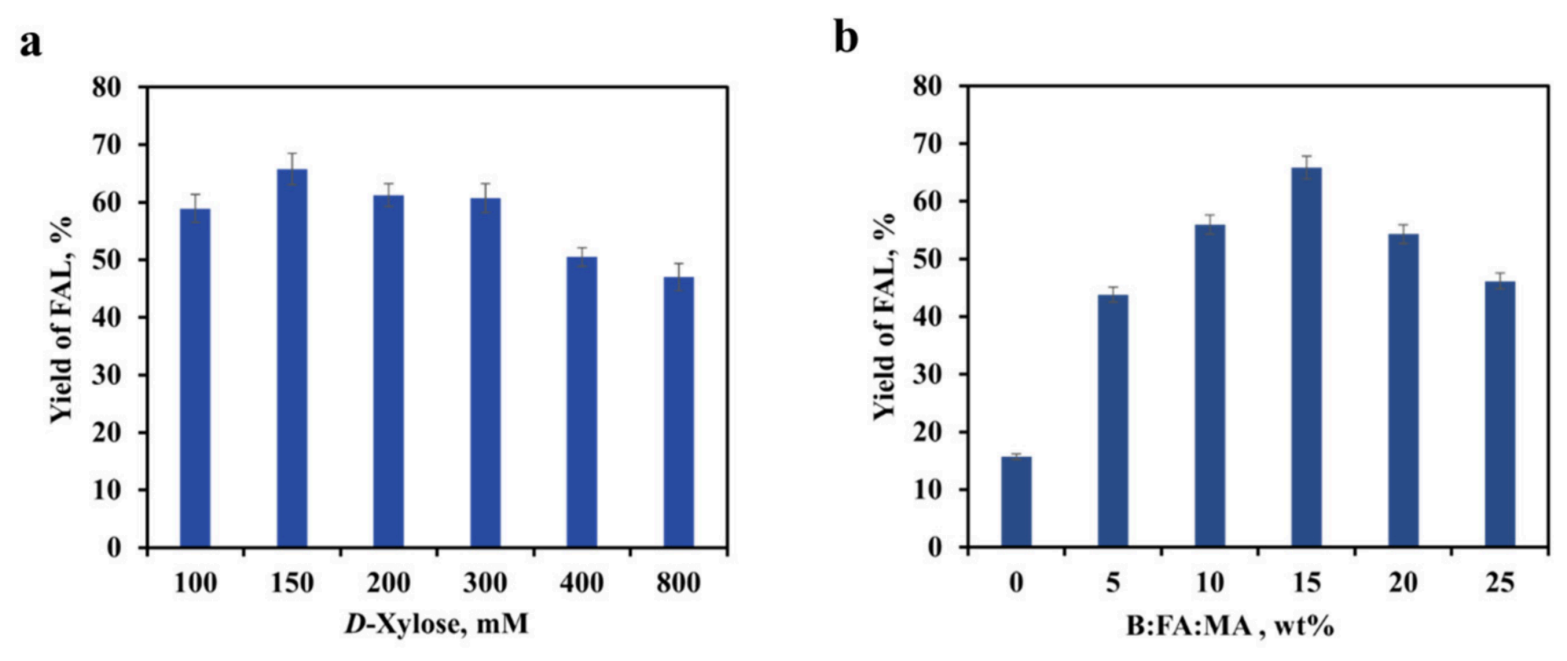
Effects of initial *D*-xylose concentration (100–800 mM) on FAL yield [Bet:FA:MA loading, 15 wt%; 170 °C; 30 min] (**a**); effect of Bet:FA:MA loading (0–25 wt%) on FAL yield in the Bet:FA:MA–water reaction medium [150 mM *D*-xylose; 170 °C; 30 min] (**b**). Data are presented as mean ± standard deviation (SD) from independent experiments (*n* = 3).

The effects of *D*-xylose loading and Bet:FA:MA loading were also investigated at 170 °C for 30 min. Increasing the initial *D*-xylose concentration from 100 to 800 mM showed that the highest FAL yield, 65.8%, was obtained at 150 mM *D*-xylose (Figure 4a). The amount of Bet:FA:MA also had a marked effect on FAL formation. As the Bet:FA:MA loading increased from 0 to 15 wt%, the FAL yield increased from 15.7% to 65.8%, whereas further increases to 20–25 wt% resulted in lower yields (Figure 4b). Thus, 15 wt% Bet:FA:MA gave the highest FAL yield among the tested loadings. Related studies have likewise shown that the composition and loading of acidic eutectic-type reaction media can influence the formation of furan products [40,41,42,43]. However, the specific reason for the decrease in FAL yield above 15 wt% was not determined in the present study, and contributions from medium acidity, individual components, and other physicochemical effects cannot be distinguished from the present data.

**Figure 5:**
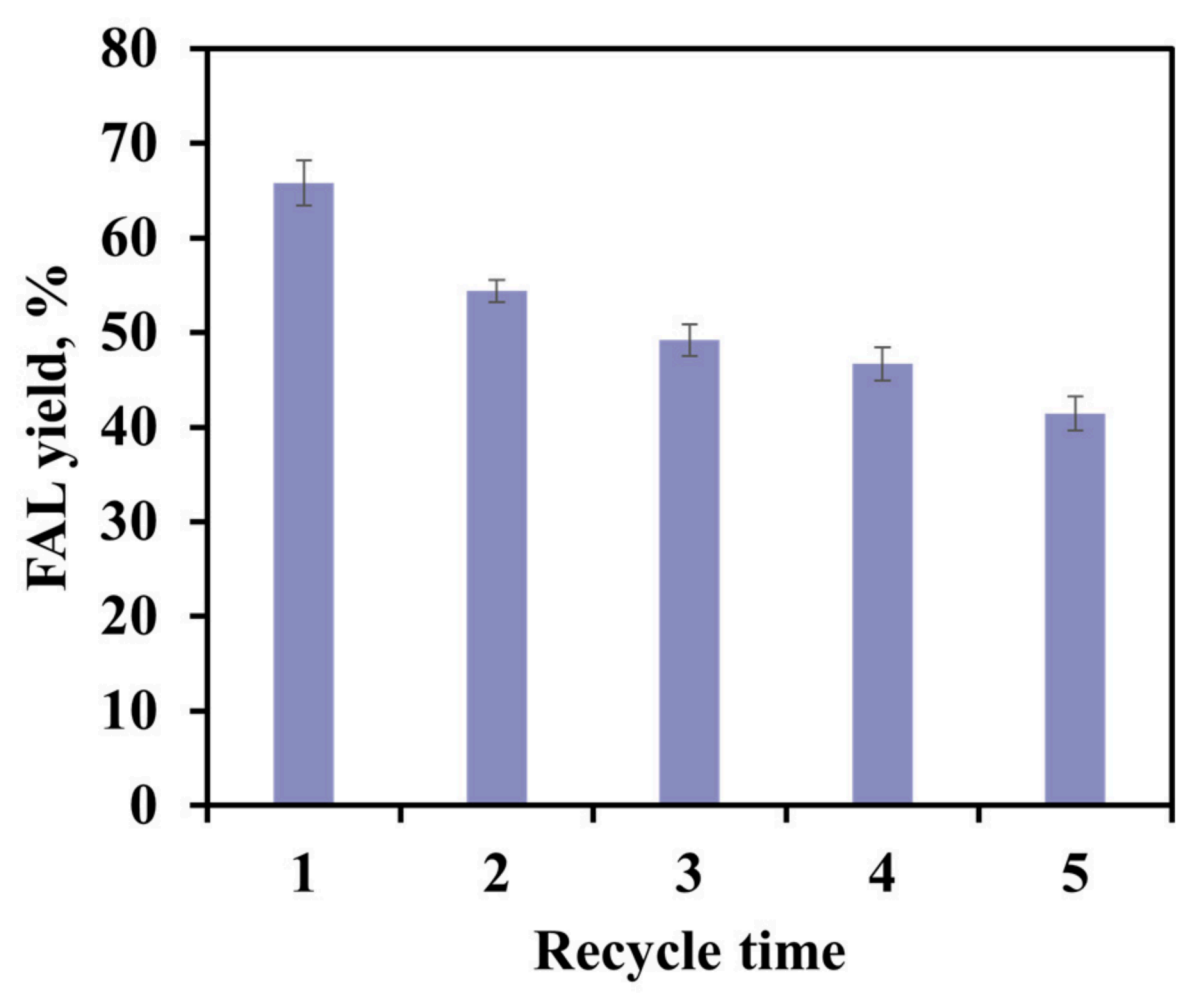
Reuse of the Bet:FA:MA–water reaction medium for *D*-xylose dehydration. Data are presented as mean ± standard deviation (SD) from independent experiments (*n* = 3).

Based on these screening experiments, Bet:FA:MA was selected as the ternary reaction medium for subsequent *D*-xylose dehydration experiments. Under the selected conditions, 1.125 g of *D*-xylose was treated with 15 wt% Bet:FA:MA at 170 °C for 30 min, giving a FAL molar yield of 65.8%. The reusability of the Bet:FA:MA–water reaction medium was further evaluated over five successive cycles. After each cycle, residual solids were removed by filtration, and FAL was extracted three times with ethyl acetate. The recovered aqueous Bet:FA:MA-containing phase was then reused for the next dehydration reaction. The FAL yield progressively decreased from 63.6% in the first cycle to 41.4% in the fifth cycle (Figure 5), indicating that the recovered reaction medium did not maintain constant catalytic performance. Several factors may contribute to this decline, including loss or redistribution of Bet, FA, and MA during product extraction and recovery, changes in water content and acidity, accumulation of dehydration-derived by-products, and possible thermal alteration of the reaction medium during repeated high-temperature treatment. Because the composition and physicochemical properties of the recovered medium were not quantitatively characterized after each cycle, the relative contributions of these factors could not be determined. Further compositional and physicochemical characterization will therefore be required to clarify the origin of the observed activity loss.

### 3.2. Construction of Recombinant EutG-GDH in E. coli Cells

**Figure 6:**
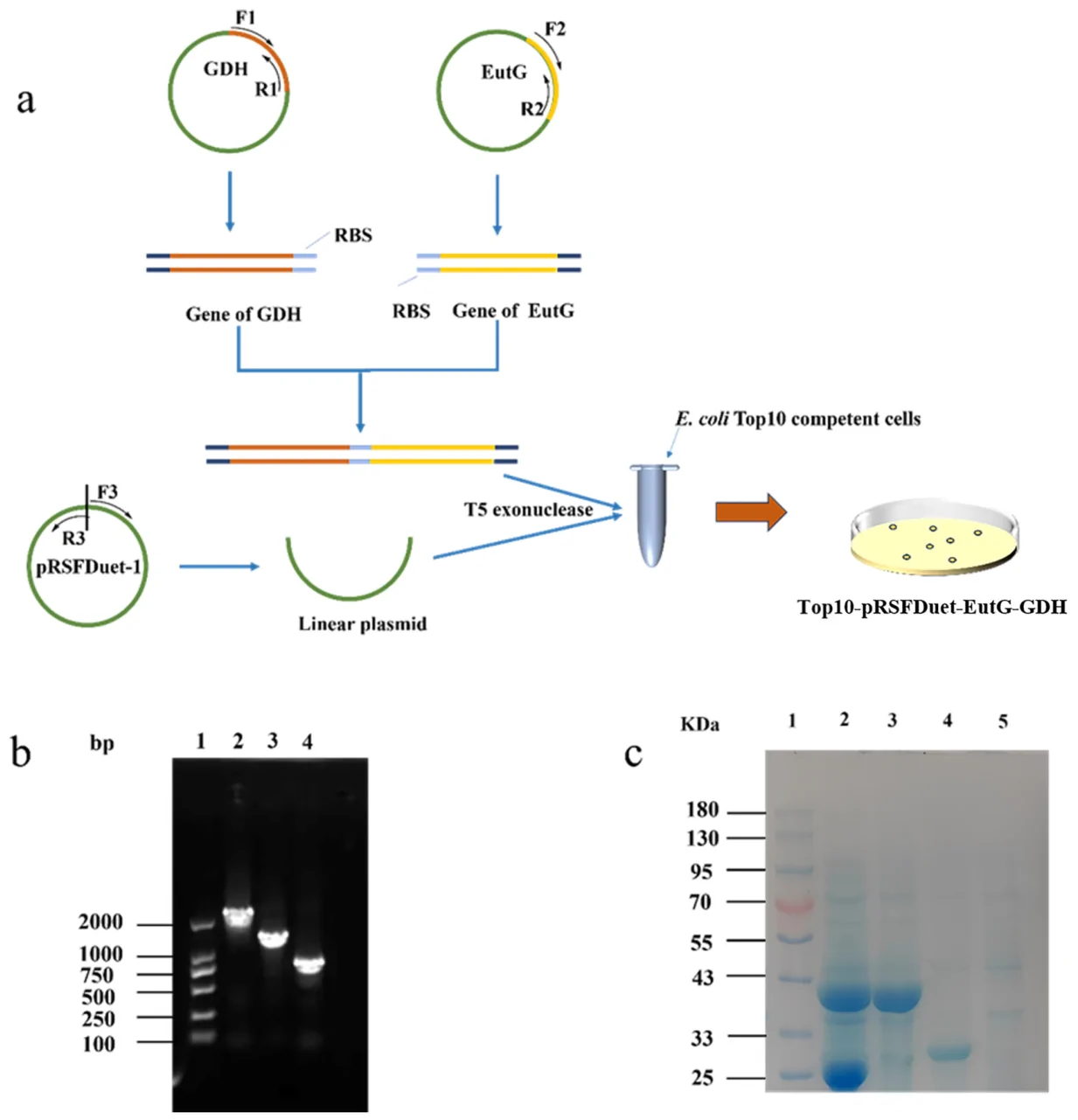
Construction process of recombinant *E. coli* EutG-GDH (**a**); analysis of the EutG-GDH gene fragment by electrophoresis. Lane 1: marker. Lane 2: EutG-GDH gene. Lane 3: EutG gene. Lane 4: GDH gene (**b**); SDS-PAGE analysis of recombinant proteins of EutG and GDH. Lane 1: protein marker; Lane 2: *E. coli* BL21 expressing pRSFDuet-EutG-GDH; Lane 3: *E. coli* BL21 expressing pRSFDuet-EutG only; Lane 4: *E. coli* BL21 expressing pRSFDuet-GDH only; Lane 5: *E. coli* BL21 with plasmid only (**c**).

To catalyze the reduction of *D*-xylose-derived FAL to FOL, recombinant EutG–GDH whole cells were constructed (Figure 6a). GDH oxidizes glucose while reducing NAD^+^ to NADH, whereas EutG catalyzes the reduction of FAL to FOL. Co-expression of EutG and GDH was confirmed by electrophoretic and SDS-PAGE analyses (Figure 6b,c). The SDS-PAGE bands (Figure 6c) indicated the molecular weight of the expressed proteins. Among them, the reductase has a molecular weight of 41.0 kDa, and GDH has a molecular weight of 28.1 kDa. The results are consistent with expectations.

Under the same reaction conditions, the EutG–GDH whole-cell system essentially completely converted 150 mM FAL within 24 h, whereas EutG-only cells afforded approximately 72% FOL yield. This improvement is consistent with a beneficial contribution of GDH co-expression to the whole-cell reduction system. However, because GDH-only and empty-vector catalytic controls and direct intracellular NADH measurements were not performed, GDH-mediated NADH regeneration is regarded here as a mechanistic interpretation rather than as directly established evidence.

### 3.3. Effects of Reaction Parameters on Whole-Cell Bioreduction

**Figure 7:**
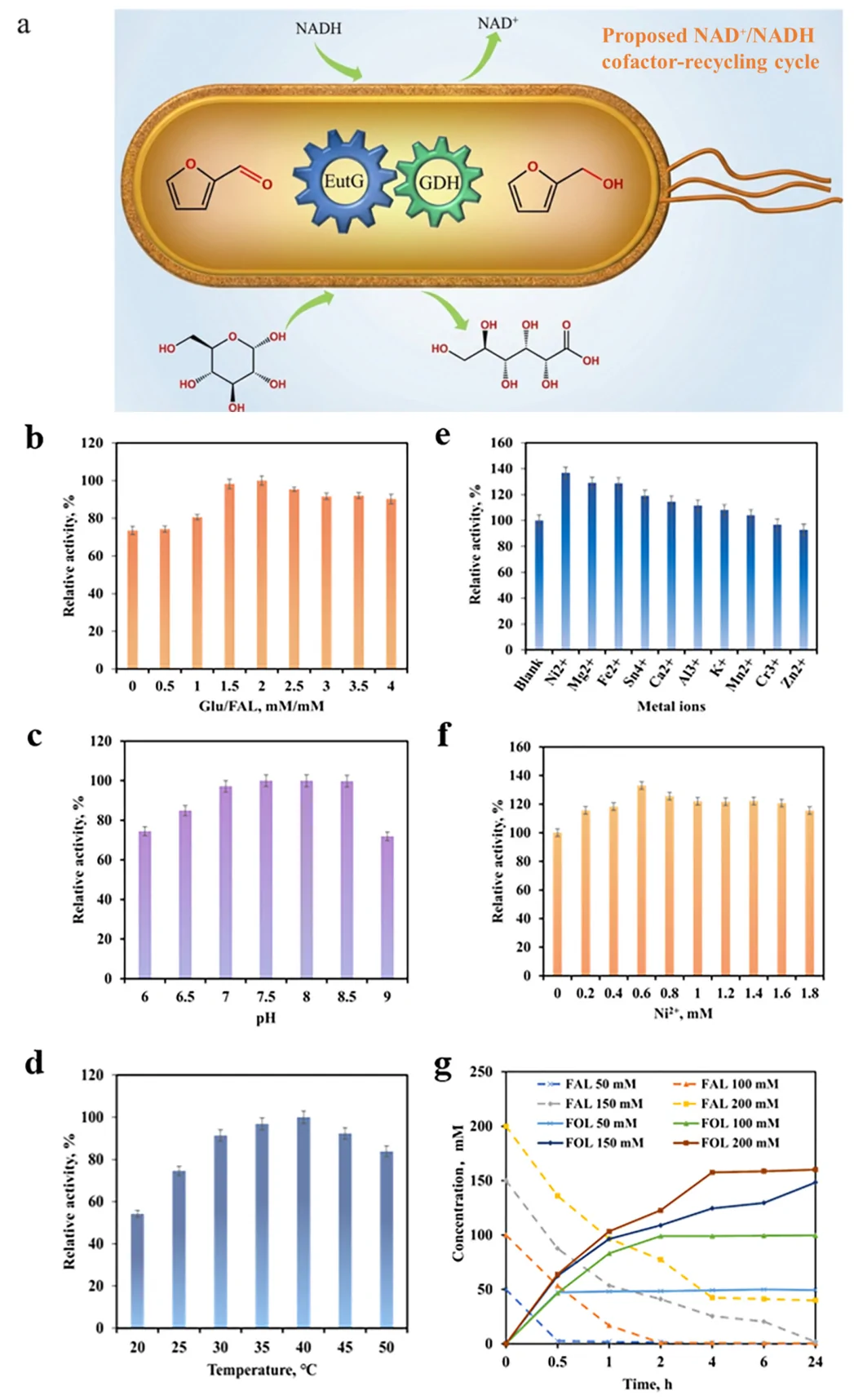
Proposed intracellular NAD^+^/NADH cofactor-recycling scheme of the EutG–GDH whole-cell system (**a**); Effects of co-substrate glucose load on the bioreduction [100 mM FAL, 35 °C, pH 6.5] (**b**); Effects of medium pH (6.0–9.0) on the bioreduction [100 mM FAL, 35 °C] (**c**); Effects of bioreaction temperature on the bioreduction at 20–50 °C [100 mM FAL, pH 7.5] (**d**); Effect of metal ions on FAL-reducing activity [100 mM FAL, 40 °C, pH 7.5] (**e**); Effect of Ni^2+^ dosage on FAL-reducing activity [100 mM FAL, 40 °C, pH 7.5] (**f**); Biological conversion of commercial FAL by whole cells (**g**). Glucose-loading and pH screening experiments were initially conducted at 35 °C. Reaction temperature was subsequently evaluated over the range of 20–50 °C, after which 40 °C was selected for the subsequent metal-ion, substrate-loading, and sequential-process experiments. Data are presented as mean ± standard deviation (SD) from independent experiments (*n* = 3).

In the process of biological reduction, the loading of co-substrate can impact the biocatalytic ability [44,45]. Using glucose as co-substrate, FAL can be biologically transformed into FOL with EutG-GDH whole cell (Figure 7a). Within the glucose loading of 2 mol glucose/mol FAL, the FAL bioreduction activity also increased with increasing glucose concentration. As the molar ratio exceeded 2:1, the biocatalytic activity of EutG-GDH whole cells decreased (Figure 7b). This was due to the fact that the viscosity of the reaction medium might increase with the improvement of co-substrate loading, thus weakening the activity of biocatalysts [46]. Accordingly, a glucose-to-FAL molar ratio of 2:1 gave the highest activity among the tested conditions and was selected for subsequent experiments. The pH value of the bioreaction medium is known to play a key role in biotransformation. The reaction pH can alter the ionization states of the enzyme and substrate [47]. Phosphate buffer solutions (100 mM) with different pH values were prepared using K_2_HPO_4_ and KH_2_PO_4_. At 35 °C, the effects of different pH values (6.0–9.0) on the whole-cell catalytic conversion of FAL to FOL by EutG–GDH were evaluated. The catalytic activity gradually increased as the pH increased from 6.0 to 7.5, whereas it decreased above pH 7.5. Similar pH-dependent behavior has been reported for other recombinant whole-cell systems catalyzing FAL reduction, with excessively acidic or alkaline conditions potentially affecting enzyme conformation and catalytic activity [22]. Among the tested pH values, the highest biocatalytic activity was observed at pH 7.5 (Figure 7c). The biological reaction temperature will affect the activity, stability, and reaction balance of biocatalysis [48,49]. Consequently, the impact of different temperatures on whole-cell FAL synthesis of FOL catalyzed by EutG-GDH was verified in a water system with pH 7.5. The results showed that the whole-cell biocatalytic activity of EutG-GDH increased significantly when the temperature increased from 20 °C to 40 °C. The whole-cell activity of EutG-GDH decreased above 40 °C. Among the tested temperatures, the highest catalytic activity was observed at 40 °C (Figure 7d). Thus, pH 7.5 and 40 °C were selected for the subsequent experiments based on the screening results.

Metal ions can influence the catalytic activity of dehydrogenase-based biocatalytic systems [50], and whole-cell bioreduction has been widely investigated for the efficient conversion of FAL to FOL [51]. Consequently, the impacts of different metal ions (K^+^, Cr^3+^, Ni^2+^, Ca^2+^, Sn^4+^, Zn^2+^, Mg^2+^, Fe^2+^, Mn^2+^ and Al^3+^) (1 mM) on whole-cell biological reduction in FAL to FOL were examined through the biotransformation with EutG-GDH whole-cells. The results showed that Ni^2+^ could promote the whole-cell catalysis of EutG-GDH, while Zn^2+^ and Cr^3+^ metal ions might inhibit the catalytic activity of cells (Figure 7e). The impacts of Ni^2+^ supplementation (0–1.8 mM) on the biological reaction of FAL reduction were further tested (Figure 7f). With the increase in Ni^2+^ content from 0 to 0.6 mM, the reducing activity was greatly augmented. In the presence of 0.6 mM Ni^2+^, the FAL-reducing activity was increased by 32% compared with the control group. As the content of Ni^2+^ increased from 0.6 mM to 1.8 mM, the promoting effect of Ni^2+^ was significantly reduced. The beneficial effect of Ni^2+^ on apparent whole-cell activity was observed experimentally, but its mechanism remains unclear and may involve effects on enzyme activity, cellular metabolism, membrane properties, or intracellular redox balance. Therefore, Ni^2+^ supplementation is regarded here as an empirical observation rather than an optimized process parameter.

The substrate loading has a crucial influence on the catalytic activity of cells [52]. The impacts of different FAL loading (50–200 mM) with co-substrate glucose (2 mol glucose/mol FAL) on the FOL synthesis with EutG-GDH whole-cell were measured in water at pH 7.5 and 40 °C. FAL at initial concentrations of ≤150 mM was essentially completely converted within 24 h. 50 mM and 100 mM FAL might be completely transformed within 2 h. For 150 mM, the conversion rate of FAL in 6 h reached 86.3%. At 200 mM FAL, conversion was 31.9% after 0.5 h. From 2 h to 6 h, the conversion rate increased from 56.3% to 79.3%. At 24 h, the conversion rate of FAL reached 80%. With the increase in FAL concentration, the catalytic ability of EutG-GDH cells gradually dropped (Figure 7g). Compared to EutG-expressing *E. coli* whole cells (Figure S3, see Supplementary Files), EutG-GDH cells have better biocatalytic ability. The improved conversion observed with EutG–GDH cells is consistent with a beneficial contribution of GDH co-expression, rather than direct proof of intracellular NADH regeneration. Overall, these experiments were intended to screen the effects of individual reaction parameters rather than to perform comprehensive process optimization. Based on the tested ranges, pH 7.5, 40 °C, and a glucose-to-FAL molar ratio of 2:1 were selected as suitable conditions for subsequent bioreduction experiments. The wet-cell loading of 0.1 g/mL used in this study is relatively high and was selected to establish the feasibility of FAL bioreduction rather than as an optimized catalyst-loading parameter. Reduction in whole-cell loading while maintaining FOL productivity should therefore be addressed in future process optimization.

### 3.4. Sequential Chemo-Biocatalytic Production of Furfuryl Alcohol from D-Xylose

**Figure 8:**
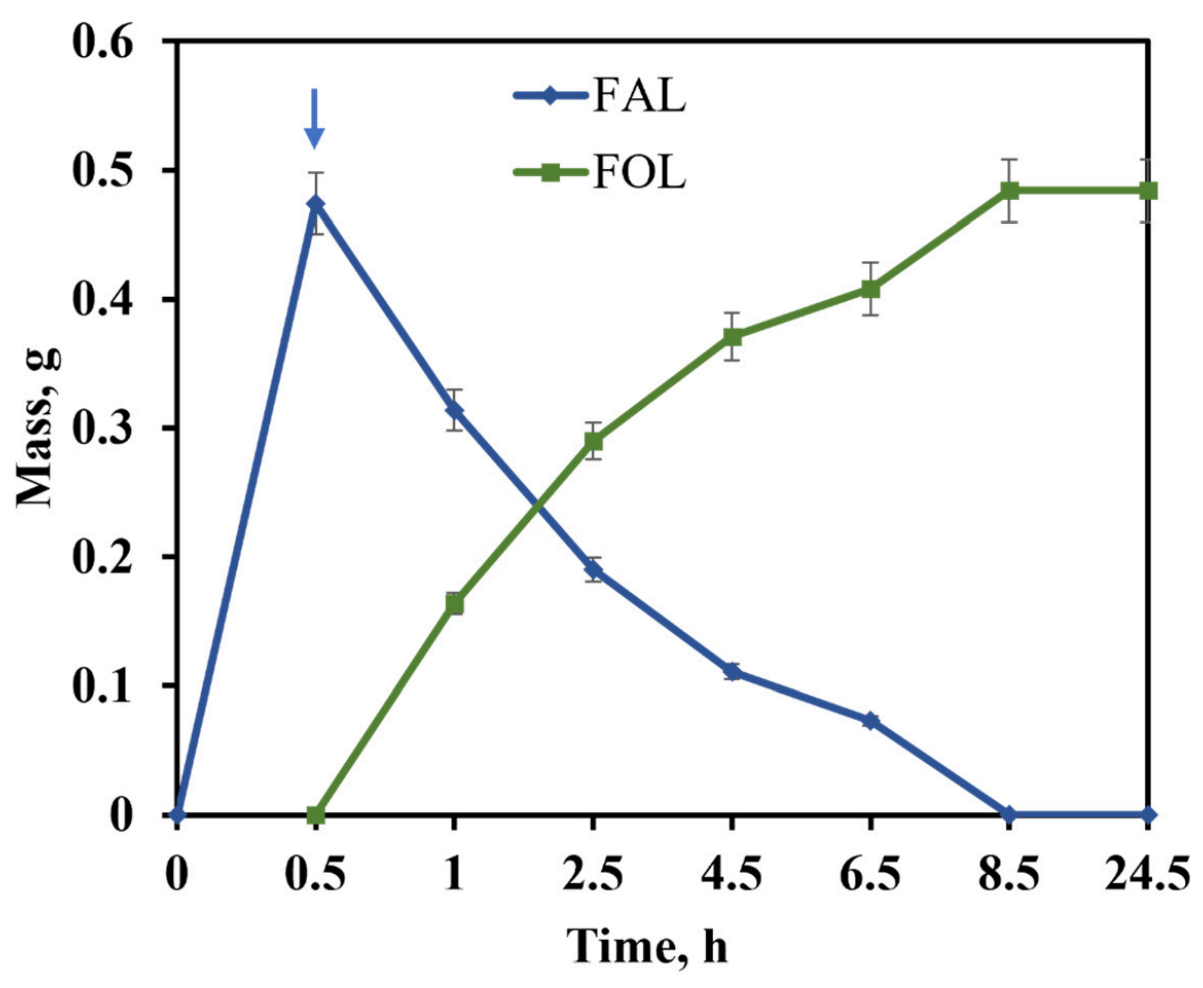
Bioreduction of *D*-xylose-derived FAL to FOL in the sequential two-stage process. Data are presented as mean ± standard deviation (SD) from independent experiments (*n* = 3).

Sequential chemical and biological approaches have been investigated for the conversion of biomass-derived carbohydrates and furan intermediates into value-added chemicals [46,53]. Related two-stage biocatalytic strategies have also been applied to the upgrading of biomass-derived aldehydes [54]. Other sequential chemo-biocatalytic systems have likewise been developed for the conversion of lignocellulosic feedstocks and furan intermediates to FOL [55,56]. Representative previously reported chemo-biocatalytic systems for FOL production are compared with the present work in Table S3 (see Supplementary Files). In the present study, Bet:FA:MA–water-mediated dehydration and EutG–GDH whole-cell reduction were combined sequentially for the conversion of *D*-xylose to FOL. A reaction mixture containing 1.125 g of *D*-xylose, 7.5 g of Bet:FA:MA, and 41.375 g of deionized water was prepared to give a total reaction mass of 50.0 g. After reaction at 170 °C for 30 min, the FAL concentration in the *D*-xylose-derived dehydration liquor reached 98.7 mM. Under these dehydration conditions, 1.125 g of *D*-xylose afforded 0.474 g of FAL, corresponding to approximately 4.93 mmol FAL and a molar yield of 65.8%. Because the resulting dehydration liquor was acidic and was not directly compatible with the whole-cell reaction conditions, an interstage conditioning step was required. The reaction liquor was adjusted to pH 7.5 and diluted from 98.7 to 30.0 mM FAL before bioreduction. EutG–GDH whole cells were then applied at a wet-cell loading of 0.1 g/mL at 40 °C, with glucose supplied at a glucose:FAL molar ratio of 2:1. Within 24 h, the conditioned 30.0 mM FAL feed was essentially completely converted to FOL, corresponding to a final FOL concentration of approximately 30.0 mM (2.94 g/L) (Figure 8). FOL was identified and quantified by HPLC through comparison with an authentic FOL standard. However, ^1^H NMR characterization of FOL isolated from the sequential two-stage process was not performed in the present study.

**Figure 9:**
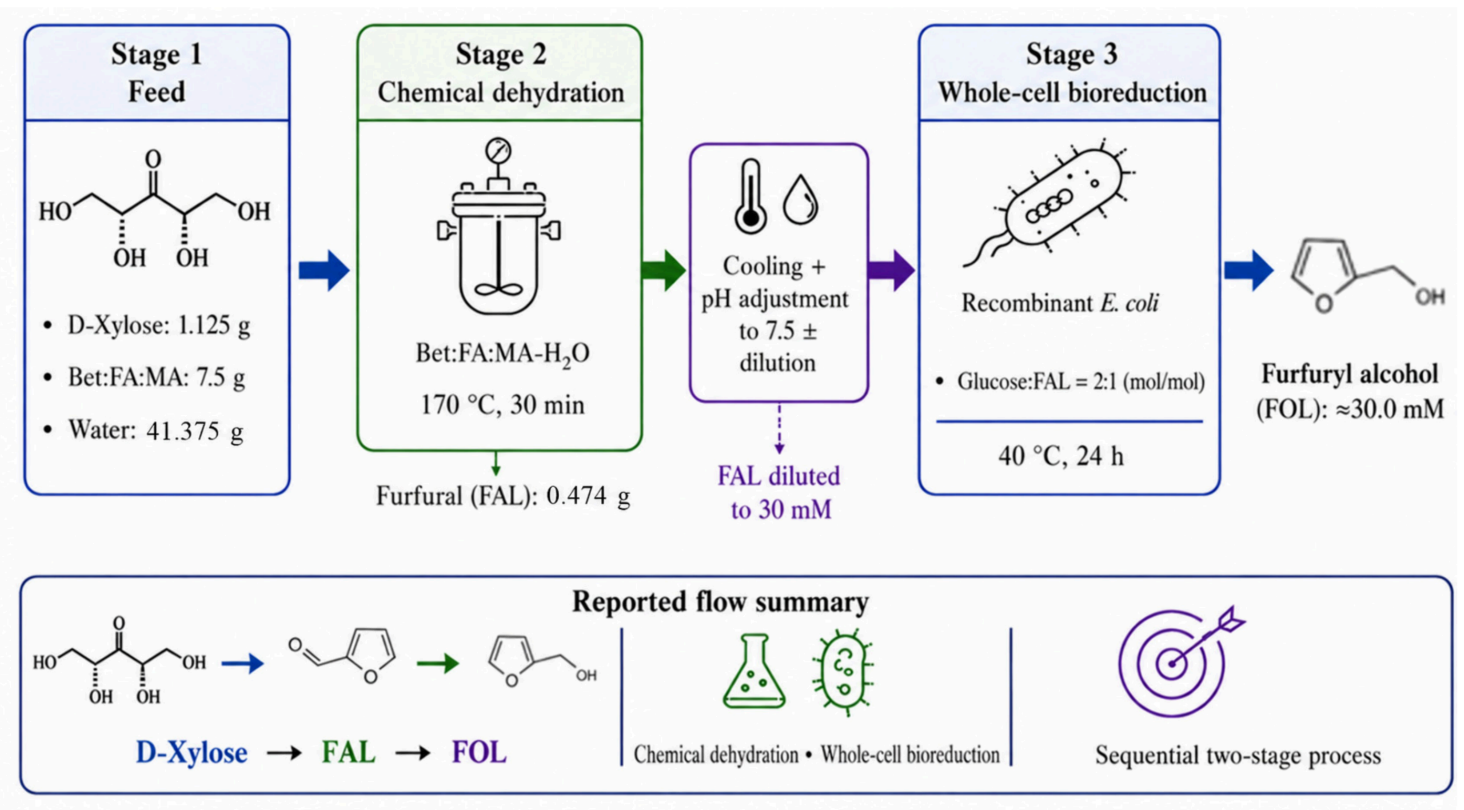
Experimentally documented quantities and process sequence from *D*-xylose dehydration to conditioned whole-cell FAL reduction.

Figure 9 summarizes the experimentally documented quantities and the sequence of operations from *D*-xylose dehydration to whole-cell FAL reduction. Because the exact liquid volume after pH adjustment and dilution was not independently recorded, an experimental overall mass or molar yield from *D*-xylose to FOL cannot be reliably calculated from the present dataset. Therefore, the 65.8% value reported above refers only to the *D*-xylose-to-FAL dehydration step and is not presented as an overall *D*-xylose-to-FOL yield. Ternary eutectic and DES-like reaction media have been investigated in biomass processing, extraction, and catalytic or biocatalytic applications, with their behavior depending strongly on composition, acidity, water content, and intermolecular interactions [57,58,59,60,61,62,63,64]. In the present study, however, the physicochemical nature of Bet:FA:MA was not independently established; therefore, the system is described here conservatively as a Bet:FA:MA–water ternary reaction medium rather than assigning the observed dehydration specifically to eutectic interactions. Biocatalysis and chemo-biocatalytic upgrading have been widely explored for the transformation of biomass-derived platform chemicals, including furfural and related aldehydes [65,66,67,68]. In parallel, conventional FAL-to-FOL conversion is commonly achieved using heterogeneous metal catalysts or catalytic transfer hydrogenation systems [69,70,71,72,73,74,75]. The present work does not seek to establish a fundamentally new cascade concept, but rather evaluates a specific sequential combination of Bet:FA:MA–water-mediated *D*-xylose dehydration and EutG–GDH whole-cell FAL reduction under laboratory-scale conditions. The results demonstrate the feasibility of this sequential two-stage combination after interstage conditioning. However, the requirement for pH adjustment and dilution of the authentic dehydration liquor indicates that compatibility between the chemical and biological stages remains a limitation of the present process.

### 3.5. Future Prospects

**Figure 10:**
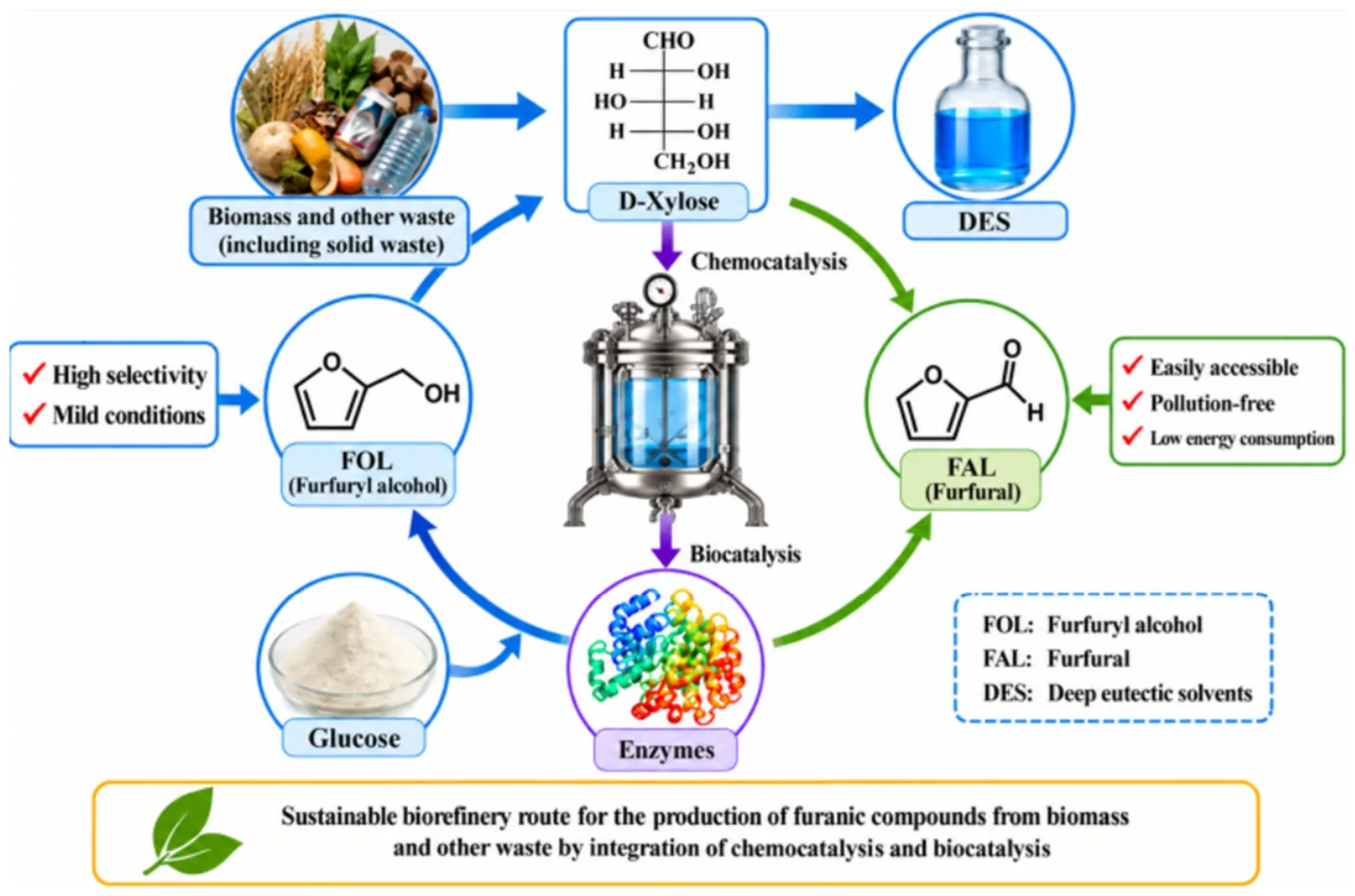
Process-flow schematic presentation of *D*-xylose valorization.

**Figure 11:**
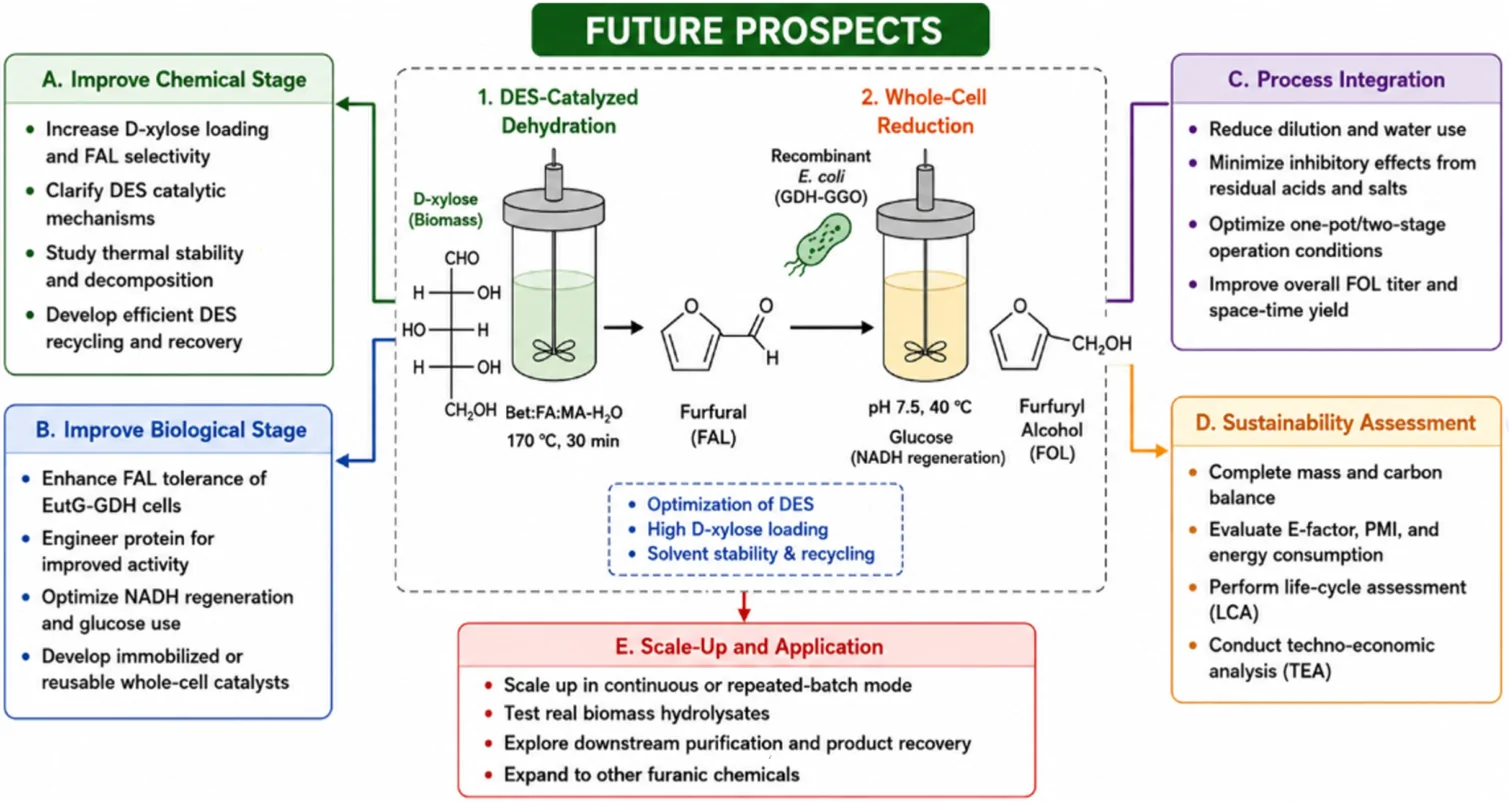
Future prospects of *D*-xylose valorization via chemobiological approach.

Future research should focus on increasing the FOL titer while reducing water, glucose, and whole-cell consumption. Improving the FAL tolerance and catalytic efficiency of EutG through protein engineering, adaptive evolution, or transporter engineering could permit direct conversion of more concentrated crude FAL streams and minimize the dilution required before bioreduction. The compatibility between the chemical and biological stages requires further quantitative evaluation and improvement. Particular attention should be given to the effects of residual formic acid, malonic acid, betaine, salts formed during pH adjustment, and FAL-derived inhibitors on cell viability, membrane integrity, EutG activity, and intracellular NADH regeneration. Immobilized or reusable whole-cell catalysts may further reduce catalyst consumption and facilitate continuous or repeated-batch operation. For the chemical stage, future work should evaluate the physicochemical properties and thermal stability of Bet:FA:MA, identify possible decomposition products, and determine whether the catalytic effect arises from formation of a genuine DES or primarily from the acidity of formic and malonic acids. Component-only controls, acid-matched controls, solvent-recycling studies, and optimization at higher *D*-xylose loadings are needed. Phase-behavior analysis, DSC, FTIR, ^1^H NMR, and pH measurements are now identified as necessary future studies for clarifying the physicochemical nature and catalytic role of the Bet:FA:MA system. Finally, a complete process assessment should quantify *D*-xylose conversion, FAL selectivity, FOL recovery, glucose consumption, gluconate formation, humin production, solvent losses, water demand, and product-isolation efficiency. Process mass intensity, E-factor, energy consumption, techno-economic analysis, and life-cycle assessment will be required to establish whether the proposed route offers genuine environmental and economic advantages over existing chemical and chemo-biocatalytic processes [76]. Future studies should determine whether such chemo-biocatalytic approaches can provide technically, environmentally, and economically competitive routes for *D*-xylose valorization (Figure 10 and Figure 11).

## 4. Conclusions

A sequential two-stage chemo-biocatalytic process combining Bet:FA:MA–water-mediated *D*-xylose dehydration with whole-cell FAL reduction was investigated. Among the six ternary formulations evaluated, Bet:FA:MA gave the highest FAL yield. Under the selected conditions of 15 wt% Bet:FA:MA, 170 °C, and 30 min, the FAL yield reached 65.8%.

A recombinant *E. coli* strain co-expressing the aldehyde reductase EutG and glucose dehydrogenase was constructed for FAL bioreduction. Under the selected conditions of pH 7.5, 40 °C, and a glucose:FAL molar ratio of 2:1, EutG–GDH whole cells essentially completely converted 150 mM commercial FAL within 24 h. The improved conversion relative to EutG-only cells was consistent with a beneficial contribution of GDH co-expression, although direct intracellular NADH measurements and a complete catalytic control set were not performed.

For the sequential process, the *D*-xylose-derived dehydration liquor was cooled, adjusted to pH 7.5, and diluted from 98.7 to 30.0 mM FAL before whole-cell bioreduction. The conditioned feed was essentially completely converted to approximately 30.0 mM FOL within 24 h. Because the exact liquid volume after pH adjustment and dilution was not independently recorded, an overall *D*-xylose-to-FOL mass or molar yield could not be reliably calculated. The present system should therefore be regarded as a laboratory-scale proof-of-concept sequential chemo-biocatalytic process. Further work should focus on reducing interstage dilution and whole-cell loading, improving compatibility with the dehydration liquor, and clarifying the physicochemical nature and catalytic role of the Bet:FA:MA reaction medium.

## Data Availability

Data are contained within the article.

## Supplementary Materials

The following supporting information can be downloaded at: https://www.mdpi.com/article/10.3390/biology15181652/s1, Figure S1. Representative HPLC chromatogram of the bioreduction of FAL by EutG–GDH whole cells under the indicated reaction conditions (40 °C, pH 7.5, glucose:FAL molar ratio = 2:1). Peaks corresponding to FAL and FOL were identified by comparison with authentic standards. Figure S2. Calibration curves for the HPLC quantification of furfural (FAL) and furfuryl alcohol (FOL). (a) Calibration curve of FAL (2-furaldehyde) over the concentration range of 0.2–1.0 mM; (b) calibration curve of FOL over the concentration range of 0.2–1.0 mM. Peak area was plotted against analyte concentration, and linear regression equations and coefficients of determination (R^2^) are shown in the corresponding panels. Figure S3. Time courses for the bioreduction of commercial FAL at different initial concentrations (50–150 mM) by EutG-expressing *E. coli* whole cells under the same reaction conditions used for comparison with the EutG–GDH system. Table S1. The nucleotide sequences of EutG and GDH encoding genes. Table S2. Primer sequences of EutG and GDH. Table S3. Comparison of the present sequential *D*-xylose (or biomass)-to-FOL process with representative previously reported chemo-biocatalytic systems.

## References

[1] Song J., Liu C., Xing J., Yang W., Ren J. (2023). Linking bioenergy production by agricultural residues to sustainable development goals: Prospects by 2030 in China. Energy Convers. Manag..

[2] Khalil M., Dincer I. (2023). Development and assessment of integrated hydrogen and renewable energy systems for a sustainable city. Sustain. Cities Soc..

[3] Ardila-Suarez C., Villegas J.P., Lins E., Ghislain T., Lavoie J.M. (2022). Waste surgical masks to fuels via thermochemical co-processing with waste motor oil and biomass. Bioresour. Technol..

[4] Aydin M., Degirmenci T. (2025). Reassessing the role of resource management on sustainable environment: Impacts of taxation, depletion, renewable energy, and globalization. Sustain. Dev..

[5] Ma B., Wang A. (2025). Exploring the role of renewable energy in green job creation and sustainable economic development: An empirical approach. Energy Strategy Rev..

[6] Weidener D., Leitner W., Domínguez P., Klose H., Grande P.M. (2020). Lignocellulose fractionation using recyclable phosphoric acid: Lignin, cellulose, and furfural production. ChemSusChem.

[7] Nasir A., Adrus N., Bohari S.P.M. (2023). The effect of alkali treatment on the cellulose extracted from rice husk as potential resources of biomass. Int. J. Nanosci..

[8] Ilanidis D., Stagge S., Jönsson L.J., Martín C. (2021). Hydrothermal pretreatment of wheat straw: Effects of temperature and acidity on byproduct formation and inhibition of enzymatic hydrolysis and ethanolic fermentation. Agronomy.

[9] Dutta S. (2021). Valorization of biomass-derived furfurals: Reactivity patterns; synthetic strategies, and applications. Biomass Convers. Biorefin..

[10] Li Q., Ren J.-Q., Li Q., Di J.-H., Ma C., He Y. (2021). Sustainable conversion of biomass-derived *D*-xylose to furfuryl alcohol in a deep eutectic solvent–water system. ACS Sustain. Chem. Eng..

[11] Li N., Zong M.-H. (2022). (Chemo)biocatalytic upgrading of biobased furanic platforms to chemicals, fuels, and materials: A comprehensive review. ACS Catal..

[12] Mittal A., Ruddy D.A., Chen X., Johnson D.K. (2023). Simultaneous dehydration of glucose and xylose present in a process-relevant biorefinery hydrolysate to furfurals using heterogeneous solid acid catalysts. Energy Fuels.

[13] Yemis O., Mazza G. (2011). Acid-catalyzed conversion of xylose, xylan and straw into furfural by microwave-assisted reaction. Bioresour. Technol..

[14] Gan P., Zhang K., Yang G., Li J., Zhao Y., Chen J. (2024). Catalytic production and upgrading of furfural: A platform compound. Int. J. Mol. Sci..

[15] Yang W., Li P., Bo D., Chang H. (2012). The optimization of formic acid hydrolysis of xylose in furfural production. Carbohydr. Res..

[16] Ayvaz A., Demirbaş S.G., Demirbaş A., Demirbaş N. (2023). One-pot multicomponent reactions in deep eutectic solvents. Curr. Org. Chem..

[17] Liu J., Li X., Row K.H. (2022). Development of deep eutectic solvents for sustainable chemistry. J. Mol. Liq..

[18] Ren S., Mu T., Wu W. (2023). Advances in deep eutectic solvents: New green solvents. Processes.

[19] Torres P., Balcells M., Cequier E., Garayoa R. (2020). Effect of four novel bio-based DES (Deep eutectic solvents) on hardwood fractionation. Molecules.

[20] Pan L., Li Q., Tao Y., Ma C., Chai H., Ai Y., He Y.-C. (2022). An efficient chemoenzymatic strategy for valorisation of corncob to furfuryl alcohol in CA:Betaine-water. Ind. Crops Prod..

[21] Annatelli M., Sánchez-Velandia J.E., Mazzi G., Pandeirada S.V., Giannakoudakis D., Rautiainen S., Esposito A., Thiyagarajan S., Richel A., Triantafyllidis K.S. (2024). Beyond 2,5-furandicarboxylic acid: *Status quo*, environmental assessment, and blind spots of furanic monomers for bio-based polymers. Green Chem..

[22] Li Y.-Y., Li Q., Zhang P.-Q., Ma C.-L., Xu J.-H., He Y.-C. (2021). Catalytic conversion of corncob to furfuryl alcohol in tandem reaction with tin-loaded sulfonated zeolite and NADPH-dependent reductase biocatalyst. Bioresour. Technol..

[23] Rosales-Calderon O., Arantes V.A. (2019). A review on commercial-scale high-value products that can be produced alongside cellulosic ethanol. Biotechnol. Biofuels.

[24] Iroegbu A.O.C., Ray S.S. (2024). On the chemistry of furfuryl alcohol polymerization: A review. J. Polym. Sci..

[25] Mishra D.K., Kumar S., Shukla R.S. (2020). Furfuryl alcohol—A promising platform chemical. Biomass, Biofuels, Biochemicals.

[26] Yan Y., Bu C., He Q., Zheng Z., Ouyang J. (2018). Efficient bioconversion of furfural to furfuryl alcohol by *Bacillus coagulans* NL01. RSC Adv..

[27] Qin L.-Z., He Y.-C. (2019). Chemoenzymatic synthesis of furfuryl alcohol from biomass in tandem reaction system. Appl. Biochem. Biotechnol..

[28] Zhang X.-Y., Xu Z.-H., Zong M.-H., Wang C.-F., Li N. (2019). Selective synthesis of furfuryl alcohol from biomass-derived furfural using immobilized yeast cells. Catalysts.

[29] Vorobyova V., Halysh V., Trus I., Skiba M., Myronyuk O., Byk M., Vasyliev G. (2026). Acidic deep eutectic solvents for pretreatment of corn stalk biomass: Effects on cellulose and lignin structure and extraction efficiency. Carbohydr. Res..

[30] Wang C., Zhao C., Zhang L. (2023). Highly efficient dehydration of fructose to 5-hydroxymethylfurfural in a green deep eutectic solvents system. React. Kinet. Mech. Catal..

[31] Yang L., Liu Y., He Y.-C., Ma C. (2023). Chemobiocatalytic transformation of biomass-derived *D*-xylose into furfuryl alcohol in a sustainable reaction system. Catal. Commun..

[32] Joshi R., Tiwari M.S. (2025). Upgradation of hemicellulose-derived furfuryl alcohol to butyl levulinate by using magnetic acidic deep eutectic solvents as catalysts. Catal. Today.

[33] Zhang S., He Y.-C., Ma C. (2024). Transformation of *D*-xylose into furfuryl alcohol via an efficient chemobiological approach in a benign deep eutectic solvent lactic acid:betaine-water reaction system. J. Mol. Liq..

[34] Fan Y., Zhang D., Zheng A., Zhao Z., Li H., Yang T. (2019). Selective production of anhydrosugars and furfural from fast pyrolysis of corncobs using sulfuric acid as an inhibitor and catalyst. Chem. Eng. J..

[35] Zhang Q., Zhou X., Xu Y. (2022). Xylooligosaccharides production from xylan hydrolysis using recyclable strong acidic cationic exchange resin as solid acid catalyst. Appl. Biochem. Biotechnol..

[36] Mankar A.R., Pandey A., Modak A., Pant K.K. (2021). Microwave mediated enhanced production of 5-hydroxymethylfurfural using choline chloride-based eutectic mixture as sustainable catalyst. Renew. Energy.

[37] Shi N., Liu Q., Cen H., Ju R., He X., Ma L. (2020). Formation of humins during degradation of carbohydrates and furfural derivatives in various solvents. Biomass Convers. Biorefin..

[38] Edumujeze D., Fournier-Salaün M.-C., Leveneur S. (2025). Production of furfural: From kinetics to process assessment. Fuel.

[39] Calderon J.C., Arora J.S., Mushrif S.H. (2022). Mechanistic investigation into the formation of humins in acid-catalyzed biomass reactions. ACS Omega.

[40] Arora S., Gupta N., Singh V. (2021). pH-controlled efficient conversion of hemicellulose to furfural using choline—Based deep eutectic solvents as catalysts. ChemSusChem.

[41] Guo H., Qi X. (2024). Deep eutectic solvents for synthesis of 5-hydroxymethylfurfural. Curr. Opin. Green Sustain. Chem..

[42] Hooshmand S.E., Kumar S., Bahadur I., Singh T., Varma R.S. (2023). Deep eutectic solvents as reusable catalysts and promoter for the greener syntheses of small molecules: Recent advances. J. Mol. Liq..

[43] Guo H., Ma X., Chen Z., Guo J., Lu J. (2025). Efficient 5-hydroxymethylfurfural production in ChCl-based deep eutectic solvents using boric acid and metal chlorides. RSC Adv..

[44] He Y.-C., Jiang C.-X., Chong G.-G., Di J.-H., Ma C.-L. (2018). Biological synthesis of 2,5-bis(hydroxymethyl)furan from biomass-derived 5-hydroxymethylfurfural by *E. coli* CCZU-K14 whole cells. Bioresour. Technol..

[45] Raczyńska A., Jadczyk J., Brzezińska-Rodak M. (2021). Altering the stereoselectivity of whole-cell biotransformations via the physicochemical parameters impacting the processes. Catalysts.

[46] Yang Q., Tang Z., Xiong J., He Y. (2022). Sustainable chemoenzymatic cascade transformation of corncob to furfuryl alcohol with rice husk-based heterogeneous catalyst UST-Sn-RH. Catalysts.

[47] Li L., Li Q., Di J., He Y., Ma C. (2023). Improved synthesis of furfurylamine from a high titer of biomass-derived furfural by a thermostable triple mutant ω-transaminase in a three-component deep eutectic solvent ChCl/Lactic acid/Malonic acid system. ACS Sustain. Chem. Eng..

[48] Xu J., Zhou S., Zhao Y., Xia J., Liu X., Xu J., He B., Wu B., Zhang J. (2017). Asymmetric whole-cell bioreduction of sterically bulky 2-benzoylpyridine derivatives in aqueous hydrophilic ionic liquid media. Chem. Eng. J..

[49] Gao R., Li Q., Chai H., He Y., Yang Z., Ma C. (2023). Synthesis of 5-hydroxymethyl-2-furfurylamine from bread waste via two-step reaction. ACS Sustain. Chem. Eng..

[50] Bulut H., Valjakka J., Yuksel B., Yilmazer B., Turunen O., Binay B. (2020). Effect of metal ions on the activity of ten NAD-dependent formate dehydrogenases. Protein J..

[51] Di J., Liao X., Li Q., He Y.-C., Ma C. (2023). Significantly enhanced bioconversion of high titer biomass-derived furfural to furfuryl alcohol by robust endogenous aldehyde reductase in a sustainable way. Green Chem..

[52] Wu C., Ma C., Li Q., Chai H., He Y.-C. (2023). Efficient production of hydroxymethyl-2-furfurylamine by chemoenzymatic cascade catalysis of bread waste in a sustainable approach. Bioresour. Technol..

[53] Feng X.-Q., Li Y.-Y., Ma C.-L., Xia Y., He Y.-C. (2020). Improved conversion of bamboo shoot shells to furfuryl alcohol and furfurylamine by a sequential catalysis with sulfonated graphite and biocatalysts. RSC Adv..

[54] Suo Y.-H., Zhang J.-Q., Qi N., Huang S.-P., Gao H., Gao L.-L., Zhang C.-F., He Y.-C., Zhang J.-D. (2024). One-pot two-stage biocatalytic upgrading of biomass-derived aldehydes to optically active β-amino alcohols via sequential hydroxymethylation and asymmetric reduction amination. Green Chem..

[55] Li Q., Gao R., Zhang Y., Zhang Y., Liu T., He Y.-C., Zheng M.-M. (2023). Enhanced upgrading of corncob to furfuryl alcohol with a novel silica-supported SO_4_^2—^TiO_2_ chemocatalyst and immobilized whole-cell biocatalyst. Green Chem..

[56] Li Y., Pan L., He Y.-C. (2023). Co-production of 2,5-dihydroxymethylfuran and furfuralcohol from sugarcane bagasse via chemobiocatalytic approach in a sustainable system. Bioresour. Technol..

[57] Ibrahim A., Tshibangu M.M., Coquelet C., Espitalier F. (2025). Ternary choline chloride-based deep eutectic solvents: A review. ChemEngineering.

[58] Yao Z., Chong G., Guo H. (2024). Deep eutectic solvent pretreatment and green separation of lignocellulose. Appl. Sci..

[59] Abdelwahed F.T., Mortada W.I., Eltabey R.M. (2025). A new surfactant-based hydrophobic deep eutectic solvent for microextraction and spectrophotometric determination of Rhodamine B and Allura red in real samples. Microchem. J..

[60] Singh V., Mittal N., Dhukia S., Atri A.K., Singh V. (2024). Novel ternary based natural deep eutectic solvents (NADES) for sustainable extraction of lignin nanoparticles from waste *Pinus roxburghii* needles: A green approach. Sustain. Chem. Pharm..

[61] Perna F.M., Vitale P., Capriati V. (2020). Deep eutectic solvents and their applications as green solvents. Curr. Opin. Green Sustain. Chem..

[62] Toleugazykyzy A., Bekbayev K., Bolkenov B., Alwazeer D., Rskeldiyev B., Kuterbekov K., Bekmyrza K., Kabyshev A., Kubenova M., Opakhai S. (2025). Comprehensive review of recent trends in the use of deep eutectic solvents for the valorization of secondary lignocellulosic biomass. Sustainability.

[63] Jančíková V., Jablonský M. (2023). The acidity values of ternary DES-like mixtures based on choline chloride, lactic acid, and dihydric alcohols. J. Mol. Liq..

[64] Hammond O.S., Bowron D.T., Edler K.J. (2017). The effect of water upon deep eutectic solvent nanostructure: An unusual transition from ionic mixture to aqueous solution. Angew. Chem. Int. Ed..

[65] Zhang X., Xu S., Li Q., Zhou G., Xia H. (2021). Recent advances in the conversion of furfural into bio-chemicals through chemo- and bio-catalysis. RSC Adv..

[66] Hanefeld U., Hollmann F., Paul C.E. (2022). Biocatalysis making waves in organic chemistry. Chem. Soc. Rev..

[67] Yu J., Sun J.W., Li N. (2025). One-pot chemoenzymatic access to a cefuroxime precursor via C1 extension of furfural. Chem. Commun..

[68] Rodríguez M.A., Rache L.Y., Brijaldo M.H., Romanelli G.P., Luque R., Martinez J.J. (2021). Biocatalytic transformation of furfural into furfuryl alcohol using resting cells of *Bacillus cereus*. Catal. Today.

[69] Lan X., Wang S., Zhang H., Zhang Y., Zhang Y., Zhang Y., Wang J., Ding D. (2024). Pd–Cu/SiO_2_ catalyzed efficient hydrogen transfer of cyclohexanol and furfural platforms into cyclohexanone and furfuryl alcohol. Fuel.

[70] Chen H., Ruan H., Lu X., Fu J., Langrish T., Lu X. (2018). Efficient catalytic transfer hydrogenation of furfural to furfuryl alcohol in near-critical isopropanol over Cu/MgO-Al_2_O_3_ catalyst. Mol. Catal..

[71] Chen J., Jia W., Yu X., Sun Y., Zhang J., Yang S., Tang X., Peng L., Zeng X., Lin L. (2023). Insight into the Co^2+^/Co^3+^ sites for the selective reduction of furfural to furfuryl alcohol. Fuel.

[72] Ramirez-Barria C., Isaacs M., Wilson K. (2018). Optimization of Ruthenium based catalysts for the aqueous phase hydrogenation of furfural to furfuryl alcohol. Appl. Catal. A Gen..

[73] Lin W., Cheng Y., Liu H., Zhang J., Peng L. (2023). Catalytic transfer hydrogenation of biomass-derived furfural into furfuryl alcohol over zirconium doped nanofiber. Fuel.

[74] Liang C., Li H., Peng M., Zhang X., Jiang Q., Cui J., Ding Y., Zhang Z.C. (2022). Co decorated low Pt loading nanoparticles over TiO_2_ catalyst for selective hydrogenation of furfural. Appl. Catal. A Gen..

[75] Ruan L., Zhu L., Zhang X., Zhou C., Alasmary F.A., Luque R., Chen B.H. (2023). A highly efficient, selective and stable PtCoNi/MWCNTs nanocatalyst for furfural hydrogenation to furfuryl alcohol under mild reaction conditions. Fuel.

[76] Woodley J.M. (2022). Ensuring the sustainability of biocatalysis. ChemSusChem.

