# Supplementary material for: Sequential Conversion of D-Xylose to Furfuryl Alcohol by Bet:FA:MA–Water Dehydration and EutG–GDH Whole-Cell Bioreduction
Source: Biology (Basel). 2026 Sep 18;15(18):1652. doi: 10.3390/biology15181652 (PMC13604293; doi:10.3390/biology15181652)
Supplement: Supplementary file 1 [file biology-15-01652-s001.zip › biology-4537478-supplementary.pdf]

Support Information

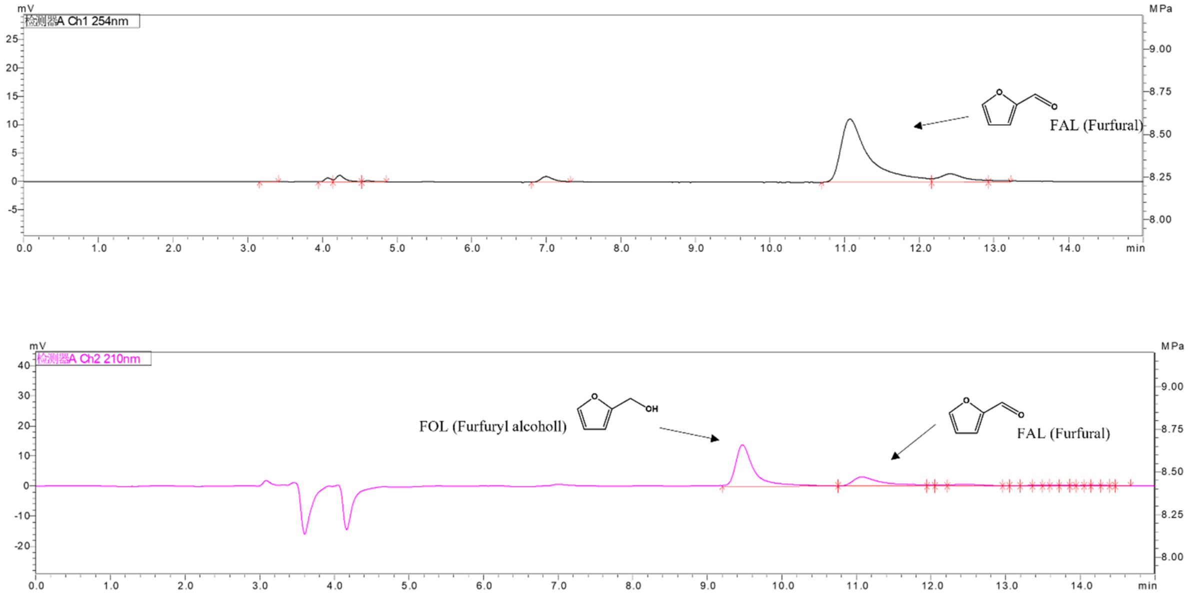

Figure S1. Representative HPLC chromatogram of the bioreduction of FAL by EutG–GDH whole cells under the indicated reaction conditions (40 °C, pH 7.5, glucose:FAL molar ratio = 2:1). Peaks corresponding to FAL and FOL were identified by comparison with authentic standards.

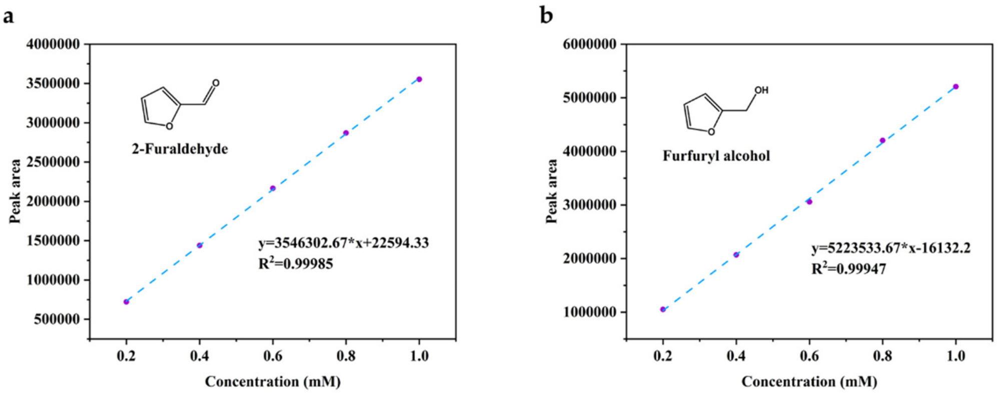

Figure S2. Calibration curves for the HPLC quantification of furfural (FAL) and furfuryl alcohol (FOL). (a) Calibration curve of FAL (2-furaldehyde) over the concentration range of 0.2–1.0 mM; (b) calibration curve of FOL over the concentration range of 0.2–1.0 mM. Peak area was plotted against analyte concentration, and linear regression equations and coefficients of determination (R^2) are shown in the corresponding panels.

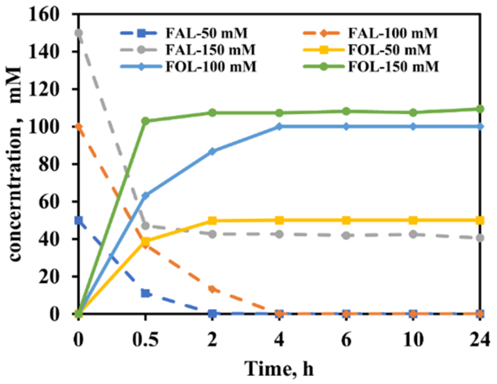

Figure S3. Time courses for the bioreduction of commercial FAL at different initial concentrations (50–150 mM) by EutG-expressing *E. coli* whole cells under the same reaction conditions used for comparison with the EutG–GDH system.

Table S1. The nucleotide sequences of EutG and GDH encoding genes.

Name	Sequence
**EutG**	ATGCAGAATGAACTGCAGACCGCCCTGTTTCAGGCATTTGATACCCTGAATCTGCAGCGTG<br>TTAAAACCTTTAGTGTTCCGCCGGTGACCCTGTGCGGTCCGGGTTCAGTGAGTAGTTGCGG<br>CCAGCAGGCCCAGACCCGTGGTCTGAAACATCTGTTTGTGATGGCAGATAGTTTTCTGCAT<br>CAGGCCGGCATGACCGCAGGCCTGACCCGTAGCCTGACCGTGAAAGGCATTGCAATGACC<br>CTGTGGCCGTGCCCGGTTGGTGAACCGTGCATTACCGATGTGTGTGCCGCCGTTGCACAGC<br>TGCGTGAAAGTGGCTGTGATGGCGTGATTGCATTTGGCGGCGGCAGCGTTCTGGATGCCGC<br>CAAAGCAGTTACCCTGCTGGTGACCAATCCGGATAGTACCCTGGCAGAAATGAGCGAAAC<br>CAGCGTGCTGCAGCCGCGCCTGCCGTTAATTGCAATTCCGACCACCGCAGGCACCGGTAGT<br>GAAACCACCAATGTGACCGTTATTATTGATGCCGTTAGTGGTCGCAAACAGGTGCTGGCAC<br>ATGCAAGTCTGATGCCGGATGTTGCAATTCTGGATGCAGCACTGACCGAAGGTGTGCCGAG<br>TCATGTGACCGCCATGACCGGTATTGATGCCCTGACCCATGCAATTGAAGCCTATAGCGCAC<br>TGAATGCAACCCCGTTTACCGATAGTCTGGCCATTGGCGCCATTGCAATGATTGGTAAAAGC<br>CTGCCGAAAGCAGTTGGTTATGGTCATGATCTGGCCGCACGCGAAAGCATGCTGCTGGCCA<br>GTTGCATGGCAGGCATGGCATTTAGCAGCGCCGGCCTGGGCCTGTGTCATGCCATGGCACA<br>TCAGCCGGGCGCCGCACTGCATATTCCGCATGGTCTGGCCAATGCAATGCTGCTGCCGACC<br>GTGATGGAATTTAATCGTATGGTGTGTCGCGAACGTTTTAGCCAGATTGGTCGCGCCCTGCG<br>CACCAAAAAAAGTGATGATCGTGATGCCATTAATGCCGTTAGTGAACTGATTGCAGAAGTG<br>GGCATTGGCAAACGCCTGGGTGATGTTGGCGCAACCAGTGCACATTATGGTGCATGGGCAC<br>AGGCCGCCCTGGAAGATATTTGTCTGCGTAGCAATCCGCGTACCGCAAGTCTGGAACAGAT<br>TGTTGGTCTGTATGCAGCCGCACAGTAA

**GDH**	ATGTATAAAGATTTAGAAGGAAAAGTAGTTGTCATAACAGGTTCATCTACCGGTTTAGGAA<br>AAGCAATGGCGATTCGTTTTGCGACAGAAAAAGCTAAAGTAGTTGTGAACTATCGTTCGAA<br>AGAAGAAGAAGCTAACAGCGTTTTAGAAGAAATTAAAAAAGTGGGCGGAGAGGCTATTGC<br>CGTCAAAGGTGATGTAACAGTTGAGTCTGATGTGATCAATTTAGTTCAATCTGCTATTAAAG<br>AATTTGGAAAGCTAGACGTTATGATTAATAACGCAGGAATGGAAAATCCGGTTTCGTCTCAT<br>GAAATGTCTTTAAGTGATTGGAATAAAGTCATTGATACGAACTTAACGGGAGCATTTTTAGG<br>CAGCCGTGAAGCGATTAAATATTTTGTGGAAAATGATATTAAGGGAACAGTTATTAACATGT<br>CGAGTGTTCACGAGAAAATTCCTTGGCCATTATTTGTTCATTACGCAGCAAGTAAAGGCGG<br>AATGAAGCTCATGACCGAAACACTTGCATTAGAATACGCTCCAAAAGGTATTCGTGTAAAT<br>AACATTGGACCGGGAGCGATTAATACACCGATTAACGCTGAGAAATTTGCTGATCCTGAGC<br>AGCGTGCAGATGTAGAAAGCATGATTCCAATGGGATACATTGGAGAGCCGGAAGAAATTGC<br>AGCGGTTGCTGCATGGCTAGCTTCTTCAGAGGCAAGTTATGTAACAGGGATTACACTCTTT<br>GCTGACGGCGGTATGACACAGTACCCATCATTCCAAGCAGGACGCGGATAA

Table S2. Primer sequence of EutG and GDH.

	Primer sequence
**EutG-F**	GCCATCACCATCATCACCACATGCAGAATGAACTGCAGACCGC
**EutG-R**	GATATATCTCCTTAGGTACCTTACTGTGCGGCTGCATACAG
**GDH-F**	GGTACCTAAGGAGATATATCATGTATAAAGATTTAGAAGGAAAAGTAGTTGTC
**GDH-R**	CTTAAGCATTATGCGGCCGCTTATCCGCGTCCTGCTTGG
**Duet-F**	GCGGCCGCATAATGCTTAAG
**Duet-R**	GTGGTGATGATGGTGATGGCTG

Table S3. Comparison of the present sequential *D*-xylose (or biomass)-to-furfuryl alcohol process with representative previously reported chemo-biocatalytic systems.

Ref.	DES composition / catalytic medium	Feedstock / xylose loading	FAL yield / titer	FOL yield / titer	Dilution before bioreduction	Reaction time (dehydration / bioreduction)	Catalyst / whole-cell loading
[10]	ChCl:Gly–H_2O (7.5 vol% DES); HCOOH 3.0 wt%	*D*-Xylose, 60.0 g/L	58.3% / ~233 mM	89.5% yield/ < 50 mMv	Yes / pH adjustment and medium adaptation required	30 min / 12 h	HCOOH, 3.0 wt%; recombinant *E. coli* CF (FDH–CgCR), 0.050 g wet cells/mL
[20]	CA:Betaine–H_2O (10:90, wt/wt)	Corncob, 60 g/L	48.2% based on xylan / 74.6 mM	95.8% yield/ < 60 mM	No additional FAL isolation reported	30 min / 2 h	CA:Betaine, 10 wt%; recombinant *E. coli* SF21
[31]	BE:BA–H_2O, BE:BA = 1:2 (mol/mol), 2.5 wt%	*D*-Xylose, 30 g/L	51.1% / 95.7 mM	99.0% yield/ < 95.7 mM	pH adjustment; 95.7 mM liquor used directly; two-fold dilution also examined	30 min / 3 h	BE:BA, 2.5 wt%; recombinant *E. coli* DCF, 0.10 g wet cells/mL
[33]	LA:Bet–H_2O; LA:Bet 10 vol% for dehydration	*D*-xylose, 0.75 g/50 mL	81.6% / 81.6 mM	95.8% yield; overall xylose-to-FOL yield 78.2%; ~ 68 mM FOL	Yes; pH adjusted to 7.0 and FAL diluted from 81.6 to 71.35 mM	30 min / 24 h	LA:Bet, 10 wt%; recombinant *E. coli* HMFOMUT, 2.859 g wet cells; glucose 0.8 mol/mol FAL
[46]	No DES; aqueous system for dehydration; MIBK–H_2O biphasic system for bioreduction	Corncob, 75.0 g/L	40.9% / 72.1 mM	100% yield; FOL productivity 0.304 g/g xylan; ~ 72 mM	Not mentioned	30 min / 2 h	UST-Sn-RH, 1.5 wt%; recombinant *E. coli KP*ADH; optimized bioreduction conditions: 0.10 g wet cells/mL and glucose/FAL = 2.5 mol/mol

This study	Bet:FA:MA–H_2O, 15 wt%	*D*-Xylose, 22.5 g/L (150 mM)	65.8% / 98.7 mM	Essentially complete conversion / ~30.0 mM FOL	Yes; pH adjusted to 7.5 and FAL diluted from 98.7 to 30.0 mM	30 min / 24 h	Bet:FA:MA, 15 wt%; recombinant *E. coli* EutG–GDH, 0.10 g wet cells/mL; glucose 2 mol/mol FAL
